# Synthesis of sulfur karrikin bioisosteres as potential neuroprotectives

**DOI:** 10.3762/bjoc.18.57

**Published:** 2022-05-16

**Authors:** Martin Pošta, Václav Zima, Lenka Poštová Slavětínská, Marika Matoušová, Petr Beier

**Affiliations:** 1 Institute of Organic Chemistry and Biochemistry, Academy of Sciences, Flemingovo nám. 2, 16610 Prague 6, Czech Republic

**Keywords:** acetylcholinesterase, butenolides, karrikin, sulfur

## Abstract

The only known sulfur-containing karrikin, 3-methyl-2*H*-thiopyrano[3,4-*b*]furan-2-one, has been recently identified as an extremely efficient neuroprotective butenolide. Herein, we report the targeted synthesis of this compound as well as new synthetic protocols toward a class of compounds derived from 2*H*-furo[2,3-*c*]pyran-2-ones (karrikins) via bioisosteric exchange of oxygen with sulfur. In particular, we present synthetic procedures toward bioisosteres of karrikins with one or two sulfur heteroatoms incorporated into the core backbone together with evaluation of their biological activity in inhibition of acetylcholinesterase.

## Introduction

Neurological disorders, especially the Alzheimer’s and the Parkinson’s diseases represent a serious problem for elderly populations worldwide. Studies of these neurodegenerative diseases led to the discovery of a deficit of acetylcholine and dopamine levels in the brain of patients suffering with the Parkinson’s and the Alzheimer’s disease, respectively. Therefore, research is focused on the discovery of new drugs protecting acetylcholine and dopamine levels via inhibition of acetylcholinesterase (AChE) and monoamine oxidase (MAO). A promising source of such novel drugs could be the smoke of burning plants that contains more than 4000 chemical species [1] and among them numerous compounds possess biological activities in humans. The psychotropic effect of smoke is well known for centuries and its inhalation has different effects depending on plant material used. For example, smoking of *Cannabis sativa* has a range of mental and physical effects including a number of therapeutic applications [2–4]. Currently, the most popular is the use of tobacco products as antidepressants based on the MAO inhibitory properties of tobacco smoke and its components [5–6]. However, tobacco and other plant-derived smoke include toxic and carcinogenic compounds which are according to WHO responsible for the death of millions of people every year [7–8]. Nevertheless, plant-derived smoke is an attractive resource containing many small molecules, many of which are unknown, that could have untapped potential in medicine. The study of the relationship between the components of plant-derived smoke and MAO and/or AChE can lead to safe and efficient drugs and drug scaffolds from a yet unexploited resource.

**Figure 1:**
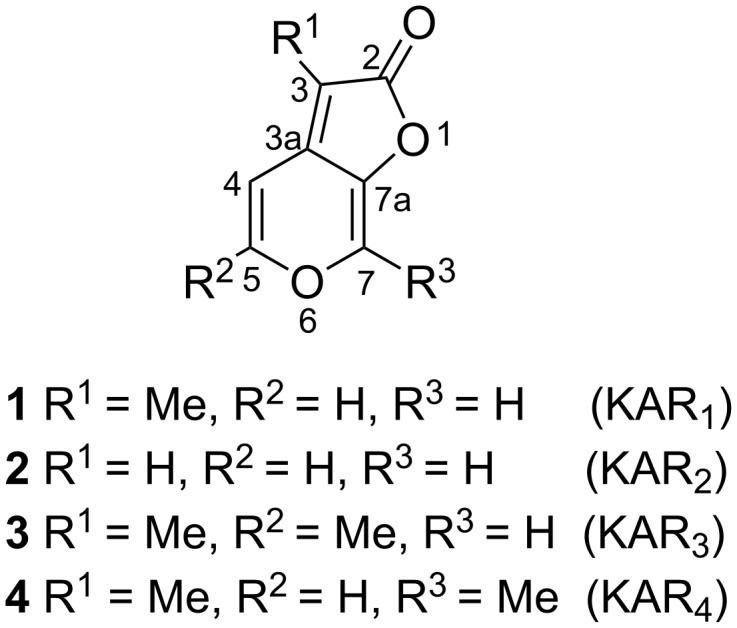
Structures of naturally occurring karrikins.

One very important bioactive constituent of smoke of burning vegetation are karrikins (Figure 1) discovered in 2004 independently by Flematti [9] and Van Staden [10]. This class of molecules has been identified as an extremely potent seed germination stimulant, promoting germination and the development of early seedling in many plant species at sub-nanomolar concentrations [11–14]. Structurally, the karrikin backbone consists of a fused pyran and a furanone ring. Each particular molecule differs in the number of methyl groups in positions C3, C5 and C7. Structures of the four most active and abundant karrikins (KAR_1_–KAR_4_) are depicted in Figure 1. The synthesis of these heterocycles is rather challenging, because the fused pyran and furanone system cannot be easily prepared by standard cyclization methods [15–17].

**Scheme 1:**
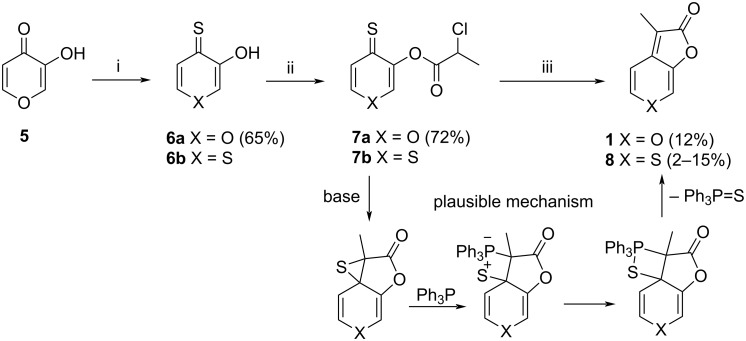
i) P_4_S_10_, THF; ii) 2-chloropropionyl chloride, Et_3_N; iii) Ph_3_P, NaOAc, Ac_2_O.

Although karrikins are extremely active plant growth regulators [14,18–19], their biological activity in humans was not investigated until recently. In 2019 a study of Naidoo et al. [20] reported naturally occurring karrikins as compounds with moderate inhibitory activity against both types of monoamine oxidases (MAO-A and MAO-B) and acetylcholinesterase, while the sulfur bioiostere 3-methyl-2*H*-thiopyrano[3,4-*b*]furan-2-one (**8**) [21] exhibited excellent activity. No targeted synthesis of **8** exists; it was isolated in low yield as a side-product in the synthesis of KAR_1_ (**1**) [21]. In this reaction sequence, thiopyranthione **6b** is the side-product in the pyranthione **6a** synthesis and both analogues were progressed to the final furan ring closure (Scheme 1). A plausible mechanism for the cyclization of compounds **7** is the Darzens reaction to episulfide, followed by Barton–Kellogg-type reaction with triphenylphosphine and elimination of triphenylphosphine sulfide. Compound **8** showed lower germination activity than KAR_1_ [22], but achieved IC_50_ values of 30 nM for MAO-B and 80 nM for AChE, respectively [20], and therefore, it represents a promising therapeutic potential against Alzheimer’s and Parkinson’s diseases.

**Figure 2:**
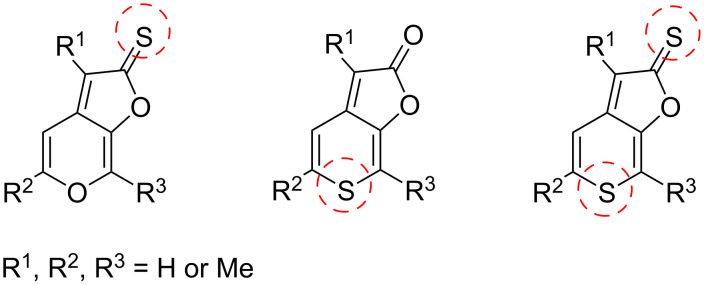
Target compounds with highlighted positions of oxygen to sulfur exchange.

The goal of this work is the development of a targeted synthesis of **8** and other sulfur analogues of karrikins in order to study the effect of bioisosteric exchange together with the effect of substitution along the karrikin backbone on the inhibitory activity against AChE. The target sulfur analogues of karrikins are shown on Figure 2.

## Results and Discussion

### Synthesis of KAR analogues with sulfur in position C2

A series of thiones, derived from the 2*H*-furo[2,3-*c*]pyran-2-thione core, differing in the number and the position of methyl groups (**9**–**12**), has been prepared from appropriate karrikins. Starting materials, karrikins KAR_1_ (**1**), KAR_3_ (**3**) and KAR_4_ (**4**) (Figure 1) were synthesized from pyromeconic acid (**5**), allomaltol (**13**) or maltol (**14**), respectively, following a published procedure [21,23], while karrikin KAR_2_ (**2**) was synthesized from ᴅ-xylose [24].

**Scheme 2:**
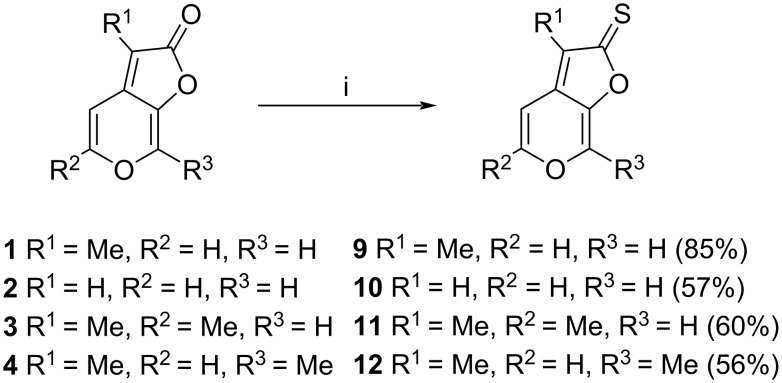
i) Lawesson’s reagent, HMDO, toluene, MW irradiation, 120 °C, 60 min.

The conversion of karrikins **1**–**4** to the corresponding C2 thiones **9**–**12** was accomplished using microwave-assisted heating with Lawesson’s reagent and hexamethyldisiloxane (HMDO) (Scheme 2) and provided the target compounds in good to high yields.

### Synthesis of KAR analogues with sulfur in position 6

**Scheme 3:**
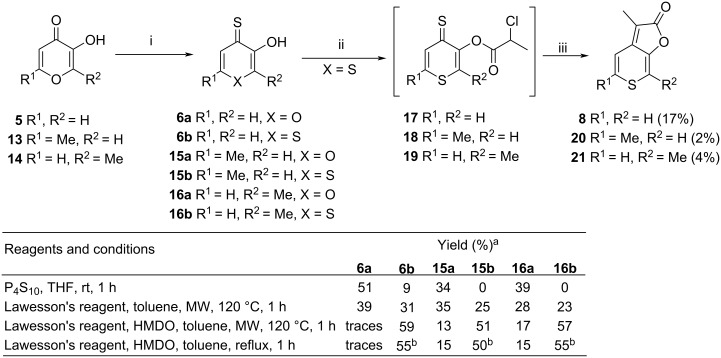
i) P_4_S_10_ or Lawesson’s reagent, see table for conditions; ii) 2-chloropropionyl chloride, Et_3_N, DCM, 0 °C; iii) AcONa, Ph_3_P, Ac_2_O, reflux. ^a^Reported yields are ^1^H NMR yields. ^b^Isolated yield.

Two different synthetic approaches were used for the preparation of C6 sulfur bioisosteres bearing a thiopyran moiety. For the synthesis of KAR_1_, KAR_3_ and KAR_4_ analogues, we proposed a synthetic strategy based on a procedure [21] employing the cyclization of appropriate esters of thiopyranthiones (Scheme 3). We expected that heterocyclic atom exchange used in the literature [25] for the synthesis of dithiomaltol **16b** directly from commercially available maltol (**14**) would proceed also with pyromeconic acid (**5**) [26] and allomaltol (**13**) [27]. Unfortunately, the treatment of hydroxypyranones **5**, **13** and **14** with Lawesson’s reagent resulted in an inseparable mixture (approx. 1:1) of pyranthiones **6a**, **15a**, **16a** and thiopyranthiones **6b**, **15b**, **16b** in low yields up to 30% in all the cases.

In order to increase the selectivity and the yields, an improved procedure [28] has been tested. An additive, HMDO applied together with Lawesson’s reagent improved significantly the yields of thiopyranthiones **6b**, **15b**, **16b** over pyranthiones **6a**, **15a**, **16a**. In contrast to previous reports on related systems [28–31], microwave heating did not improve the yields; therefore, conventional heating was used in our case.

Generally, **5** is the most reactive among the substrates and the experiments proved that only **5** and not **13** or **14** can be partially converted to thiopyranthione **6b** in the presence of P_4_S_10_ without heating. This observation explains the formation of side-product **8** in the original Flematti’s synthesis [21] (Scheme 1).

With thiopyranthiones **6b**, **15b**, **16b** in hand, we carried out the esterification step with 2-chloropropionyl chloride, followed by intramolecular cyclization which provided the desired compounds **8**, **20** and **21** albeit in low yields.

**Scheme 4:**
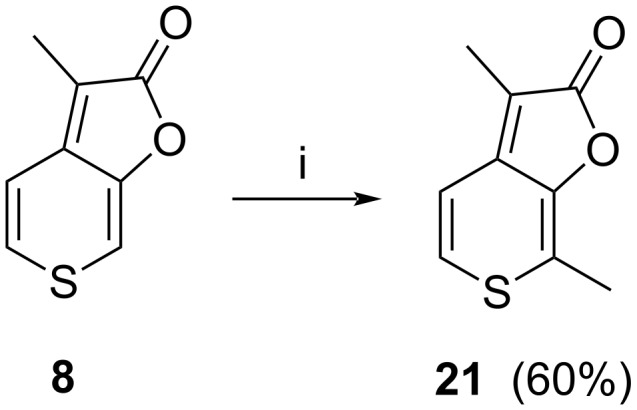
i) LiHMDS, MeI, THF, −78 °C.

Due to the extremely low isolated yield of compounds **20** and **21**, we looked for an alternative more efficient procedure. In 2008 Sun et al. [32] described a method of karrikin alkylation in position 7 via direct metalation with lithium bis(trimethylsilyl)amide (LiHMDS), followed by the addition of an alkyl halide. Application of this method to **8** provided the target molecule **21** in good yield (Scheme 4). It has to be mentioned that the metalation proceeds exclusively at C7, and thus cannot be used for the preparation of **20** via alkylation at C5.

Our attempt to prepare the desmethyl analogue **26** through esterification of **6b** with chloroacetyl chloride followed by intramolecular cyclization stayed unrewarded due to the lack of reactivity in the cyclization step.

**Scheme 5:**
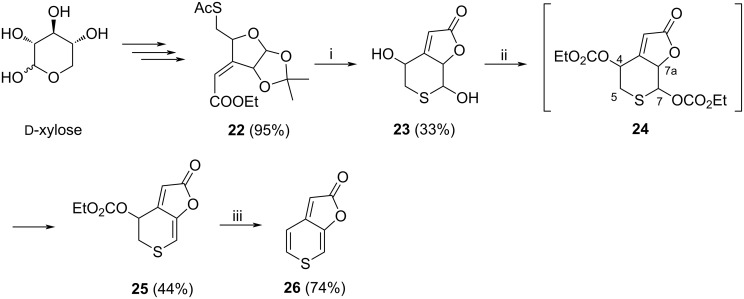
i) a) NaOH, MeOH/H_2_O, rt, Amberlyst 15 [H^+^], b) AcOH 70% aq, reflux, 2 h; ii) a) EtOCOCl, pyridine, b) Et_3_N, CH_2_Cl_2_; iii) (Ph_3_P)_4_Pd, BSA, THF.

In order to overcome this problem a new strategy was developed. We combined the synthetic protocol of Goddard-Borger [24] for the preparation of **2** using the Xavier’s procedure [33] towards 5-thiopyranose-fused butenolides and the reaction pathway is outlined in Scheme 5.

The synthesis of the key intermediate butenolide **23** was accomplished starting from easily available ᴅ-xylose, following a published multistep procedure [33]. The synthesis of target compound **26** was accomplished in two steps by double esterification and elimination of **23**. Dicarbonate **24** partially eliminated over C7–C7a to give **25** upon aqueous workup. In order to maximize the yield, the organic fraction containing a mixture of **24** and **25** was treated with triethylamine in order to obtain the eliminated product exclusively. The second elimination over C4–C5 resulted in a low yield of **26** (41%) (not shown in Scheme 5) and a high (Ph_3_P)_4_Pd catalyst load (10%) was necessary, despite the fact that allylic carbonates are good substrates in these Tsuji–Trost eliminations [34]. However, in the presence of *N,O*-bis(trimethylsilyl)acetamide (BSA) a standard catalyst loading (4 mol %) was sufficient to obtain **26** in a good yield (74%, Scheme 5).

### Synthesis of KAR analogues with sulfur in positions C2 and 6

**Scheme 6:**
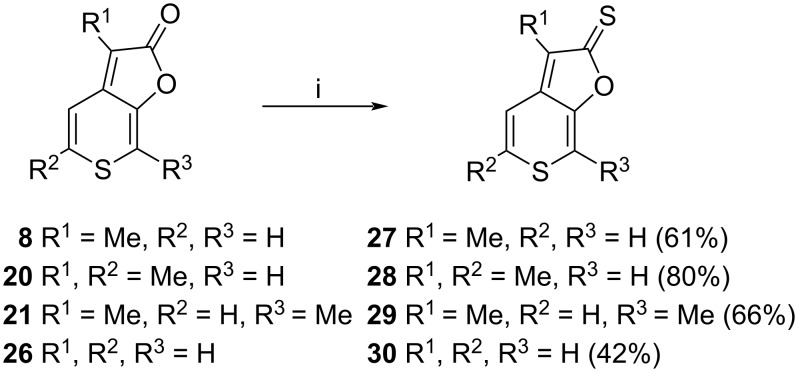
i) Lawesson’s reagent, HMDO, toluene, MW irradiation (120 °C), 60 min.

2*H*-Thiopyrano[3,4-*b*]furan-2-thione derivatives **27**–**30** were prepared from **8**, **20**, **21**, and **26** using microwave heating with Lawesson’s reagent and HMDO (Scheme 6). Thionation provided the title compounds in good to high yields.

### Biochemical study – AchE inhibition

The compounds were further tested for their ability to inhibit AChE in vitro [24]. Based on the data by Naidoo et al. [20], where KAR_3_ and sulfur-substituted karrikin (**8**) showed significant (submicromolar) inhibition effects on AChE, comparable activity was expected in the current series of compounds. However, no significant activity was observed. At 10 µM screening concentration the most active analogues (**28** and **29**) only reached approx. 15% inhibition which corresponds to the expected IC_50_ values in high micromolar range (Table 1).

**Table 1:** Inhibition of acetylcholine esterase by the synthesized analogues.

Compound	AChE residual activity at 10 µM (% of untreated)	AChE IC_50_ (µM)

none	100 ± 3	
galanthamine	16 ± 1	1.5 ± 0.5
**1**	–	477 ± 0.12 [20]
**3**	–	0.37 ± 0.02 [20]
**8**	–	0.08 ± 0.006 [20]
**9**	95 ± 8	n.d.
**10**	99 ± 3	n.d.
**11**	93 ± 3	n.d.
**12**	91 ± 2	n.d.
**20**	90 ± 2	n.d.
**21**	91 ± 2	n.d.
**26**	99 ± 0.4	n.d.
**27**	97 ± 5	n.d.
**28**	85 ± 1	n.d.
**29**	86 ± 2	n.d.
**30**	92 ± 1	n.d.

n.d. - not determined, IC_50_ >> 10 µM		

## Conclusion

In conclusion, efficient synthetic protocols for the synthesis of sulfur analogues of karrikin were developed. The original synthetic methodology for the preparation of analogues of karrikins with one sulfur atom in position C2 or position 6, or two sulfur atoms in both positions was improved; however, observed biological activities towards AChE were rather low.

## Supporting Information

File 1 Experimental part, compound characterization and copies of NMR spectra.
