# Supplementary material for: Synthesis of sulfur karrikin bioisosteres as potential neuroprotectives
Source: Beilstein J Org Chem. 2022 May 16;18:549–54. doi: 10.3762/bjoc.18.57 (PMC9127252; doi:10.3762/bjoc.18.57)
Supplement: File 1 — Experimental part, compound characterization and copies of NMR spectra. [file Beilstein_J_Org_Chem-18-549-s001.pdf]

BEILSTEIN JOURNAL OF ORGANIC CHEMISTRY

Supporting Information

for

Synthesis of sulfur karrikin bioisosteres as potential neuroprotectives

Martin Pošta, Václav Zima, Lenka Poštová Slavětínská, Marika Matoušová and Petr Beier

Beilstein J. Org. Chem. **2022,** *18,* 549–554. doi:10.3762/bjoc.18.57

Experimental part, compound characterization and copies of NMR spectra

License and Terms: This is a supporting information file under the terms of the Creative Commons Attribution License (https://creativecommons.org/licenses/by/4.0). Please note that the reuse, redistribution and reproduction in particular requires that the author(s) and source are credited and that individual graphics may be subject to special legal provisions.
The license is subject to the *Beilstein Journal of Organic Chemistry* terms and conditions: (https://www.beilstein-journals.org/bjoc/terms)

Experimental

Biochemistry

Acetylcholine esterase (AChE) assay

Determination of AChE activity was based on the original method by Ellman [1] adapted for 384 well plate format. The plates were first pre-spotted with 20 nL of 10 mM DMSO solution of the compound with the use of ECHO 550® liquid handler (to reach 10 µM final concentration). Master mix solution was prepared alongside and kept on ice until dispensed in the wells. The mix consisted of 1.5 mM acetylthiocholine iodide (ATCI), 1.5 mM 5,5-dithiobis(2-nitrobenzoic acid) (DTNB) and 30 mU/mL AChE from *Electrophorus electricus* (Sigma-Aldrich, Germany) in 50 mM TRIS-Cl buffer, pH 7.5, containing 0.04% BSA, 50 mM NaCl and 10 mM $MgCl_2$. 20 µL of the master mix was then dispensed into the wells of a transparent flat-bottom 384-well plate (Nunc). The microplate was briefly but vigorously mixed on an orbital shaker and placed into a Tecan SPARK microplate reader where the absorbance at 405 nm was continuously monitored for 25 min (linear range). In parallel, the no-enzyme control reaction was run to exclude interference of the compounds with the assay. The background value was subtracted from the signal of the enzyme-containing wells. Enzyme inhibition was expressed as a signal percentage compared to an uninhibited reaction at t = 25 min: % residual activity = (A_{405} sample/A_{405} control) × 100. Galanthamine was used as a positive control. Where applicable (residual activity < 75% at 10 µM), AChE inhibitory data were analysed with the use of Prism 9 (GraphPad Inc., San Diego, USA). The values were measured in triplicates, and the results are presented as means ± SD.

Chemistry

Nuclear magnetic resonance spectra (^{1}H and ^{13}C) were recorded on a Bruker Avance III HD 400 (400.1 and 100.6 MHz), Bruker Avance III HD 400 Prodigy (401.0 MHz and 100.8 MHz) and a Bruker Avance III HD 500 spectrometer (500.0 and 125.7 MHz), in acetone-d_6 (referenced to the residual solvent signal 2.05 ppm for ^{1}H and 29.84 ppm for ^{13}C) or $CDCl_3$ (referenced to the residual solvent signal 7.26 ppm for ^{1}H and 77.0 ppm for ^{13}C). Complete assignment of all NMR signals was performed using a combination of 2D-NMR correlation experiments (H,H-COSY, H,C-HSQC and H,C-HMBC). Chemical shifts (δ) are given in ppm and coupling constants (J) in

Hz. GC–MS spectra were recorded on an Agilent 7890A gas chromatograph coupled with a 5975C quadrupole mass-selective electron impact (EI) detector (70 eV). High-resolution mass spectra were recorded using a LTQ Orbitrap XL system (Thermo Fisher Scientific). FTIR spectra were recorded on a spectrometer Nicolet 6700 (Thermo Scientific, USA) using a standard MIR source, KBr beamsplitter, and DTGS detector which was purged with nitrogen. All samples were measured in transmission mode (64 scans, 2 cm^{-1} spectral resolution, Happ-Genzel apodization function) as films prepared from dissolved samples in $CHCl_3$. Melting points were determined using a Kofler hot-stage apparatus and are uncorrected. TLC analysis was carried out using commercial Sigma-Aldrich aluminium plates coated with silica-gel with fluorescent indicator F254s. Spots were visualized under UV and developed in permanganate stain.
Microwave experiments were carried out in CEM Discover microwave synthesizer in closed vessel equipped with stirring bar (300 W). All syntheses were carried out under an argon or nitrogen atmosphere in anhydrous solvents, which were dried with sodium and benzophenone and distilled prior to use unless stated otherwise.

Synthesis of KAR analogues with sulfur in position 2

Karrikins KAR_1, KAR_3 and KAR_4 were prepared from pyromeconic acid, allomaltol or maltol, respectively, following published procedures [2,3]. Karrikin KAR_2 was synthesized from D-xylose following a published multistep protocol [4].

General procedure:

Karrikin (0.50 mmol, 1 equiv), Lawesson´s reagent (202.2 mg, 0.50 mmol, 1 equiv) and HMDO (0.28 mL, 0.65 mmol, 1.3 equiv) were suspended in anhydrous toluene (5 mL) and heated and stirred in microwave reactor (300 W) for 60 minutes at 120 °C. After cooling, the solvent was removed under vacuum. Purification of the solid residue by column chromatography (SiO_2, DCM) afforded title compound that was re-crystallized from pentane.

*3-Methyl-2H-furo[2,3-c]pyran-2-thione (**9**)*
Yield: 70.6 mg (85%), yellow solid, mp 156-157 °C
^{1}H NMR (500 MHz, Acetone-d_6): 2.05 (s, 3H, CH_3); 7.05 (dm, 1H, $J_{4,5}$ = 5.4 Hz, H-4); 7.88 (d, 1H, $J_{5,4}$ = 5.4 Hz, H-5); 8.25 (s, 1H, H-7).

^{13}C NMR (125.7 MHz, Acetone-d_6): 9.86 (CH_3-3); 105.55 (CH-4); 118.57 (C-3); 130.45 (CH-7); 140.52 (C-3a); 149.70 (C-7a); 150.89 (CH-5); 203.44 (C-2).

HRMS: (APCI) m/z calculated for $C_8H_7O_2S$ $[M+H]^+$ 167.01613, found 167.01607.

IR: 3086(m),3075(m), 2917(m) 1650(m), 1593(m), 1535(m), 1398(m), 1342(m), 1293(m), 1146(m), 999(m),800(s).

*2H-Furo[2,3-c]pyran-2-thione (**10**)*

Yield: 43.4 mg (57%), yellow solid, mp 176-177 °C

^1H NMR (600.1 MHz, Acetone-d_6): 6.31 (d, 1H, $J_{3,7}$ = 1.4 Hz, H-3); 7.11 (d, 1H, $J_{4,5}$ = 5.3 Hz, H-4); 7.95 (d, 1H, $J_{5,4}$ = 5.3 Hz, H-5); 8.34 (d, 1H, $J_{7,3}$ = 1.4 Hz, H-7).

^{13}C NMR (150.9 MHz, Acetone-d_6): 106.34 (CH-4); 111.38 (CH-3); 131.29 (CH-7); 145.47 (C-3a); 151.34 (C-7a); 151.97 (CH-5); 203.14 (C-2).

HRMS: (APCI) m/z calculated for $C_7H_5O_2S$ $[M+H]^+$ 153.0048, found 153.00041.

IR: 3097(m),3032(m), 1649(m), 1576(m), 1530(m), 1390(m), 1354(m), 1122(s),980(m), 878(m),828(s).

*3,5-Dimethyl-2H-furo[2,3-c]pyran-2-thione (**11**)*

Yield: 54.1 mg (60%), orange solid, mp 171-173 °C

^1H NMR (500 MHz, Acetone-d_6): 2.00 (s, 3H, CH_3-3); 2.40 (d, 3H, $J_{CH3,4}$ = 0.7 Hz, CH_3-5); 6.82 (bq, 1H, $J_{4,CH3}$ = 0.7 Hz, H-4); 8.18 (s, 1H, H-7).

^{13}C NMR (125.7 MHz, Acetone-d_6): 9.80 (CH_3-3); 19.85 (CH_3-5); 102.70 (CH-4); 117.24 (C-3); 130.03 (CH-7); 142.36 (C-3a); 148.99 (C-7a); 161.45 (C-5); 203.50 (C-2).

HRMS: (ESI+) m/z calculated for $C_9H_9O_2S$ $[M+H]^+$ 181.03178, found 181.03180.

IR: 3084(m),3054(m), 2951(m),2922(m), 2854(m), 1661(s), 1602s), 1557(m),1468(m), 1382(s), 1332(s), 1211(m), 1159(s), 980(m), 855(m),837(s).

*3,7-Dimethyl-2H-furo[2,3-c]pyran-2-thione (**12**)*

Yield: 50.5 mg (56%), orange solid, mp 177-178 °C

^1H NMR (500 MHz, Acetone-d_6): 2.00 (s, 3H, CH_3-3); 2.40 (d, 3H, $J_{CH3,4}$ = 0.7 Hz, CH_3-5); 6.82 (bq, 1H, $J_{4,CH3}$ = 0.7 Hz, H-4); 8.18 (s, 1H, H-7).

^{13}C NMR (125.7 MHz, Acetone-d_6): 9.99 (CH_3-3); 14.49 (CH_3-7); 105.20 (CH-4); 118.23 (C-3); 140.49 (C-3a); 140.97 (C-7); 146.18 (C-7a); 150.69 (CH-5); 202.56 (C-2).

HRMS: (APCI) m/z calculated for $C_9H_9O_2S$ $[M+H]^+$ 181.03178, found 181.03174.

IR: 3092(m),3044(m), 2922(m), 2851(m), 1681(s), 1597(s), 1534(m),1468(m), 1350(s), 1248(m), 1161(s), 977(m), 848(m),819(m).

Synthesis of KAR analogues with sulfur in position 6

General procedure:

i) A hydroxy-pyranone (40.0 mmol, 1 equiv), Lawesson´s reagent (17.80 g, 44.0 mmol, 1.1 equiv) and HMDO (9.35 mL, 44.0 mmol, 1.1 equiv) were suspended in 50 mL of anhydrous toluene under nitrogen atmosphere and refluxed for 1.5 h. After cooling, the reaction mixture was filtered over a short silica plug eluting with toluene. Purification by column chromatography (SiO_2, gradient 5–15% of EtOAc in petroleum ether 40–60) afforded the product as an orange solid.

ii) A solution of 2-chloropropionyl chloride (1.75 mL, 18 mmol, 1.2 equiv) in DCM was added dropwise to a stirred solution of hydroxy-thiopyranthione (15 mmol, 1 equiv) and Et_3N (3.13 mL, 22.5 mmol, 1.5 equiv) in DCM at 0 °C. The reaction mixture was stirred for 30 min and allowed to warm to laboratory temperature. The volume of DCM was reduced to one half and the reaction mixture was filtered over a short plug of silica with DCM. The obtained dark red solid product was used for the next step without further purification.

iii) A solution of ester (1 equiv) in acetic anhydride was added dropwise over 15 minutes to the refluxing mixture of anhydrous sodium acetate (3 equiv) and triphenylphosphine (1.1 equiv) in acetic anhydride. The mixture was heated for 30 minutes and allowed to cool. The dark reaction mixture was poured into ice/water and stirred until one phase was formed and neutralized by the addition of $NaHCO_3$. The solution was filtered and extracted with dichloromethane. The organic extract was washed with brine, dried over $MgSO_4$, filtered and evaporated under reduced pressure. Purification by column chromatography (SiO_2, gradient 0–5% of acetone in petroleum ether 40–60) afforded pure product **8** as a yellow solid, while compounds **20** and **21** were isolated impure containing up to 20% of KAR_3 (**3**) or KAR_4 (**4**) respectively. Separation of the two products was achieved by reversed-phase HPLC (C18 MAG5, 250 × 25 mm, 7 µm, 20% MeCN in H_2O mobile phase). The product was

extracted with dichloromethane (3 × 15 mL), the combined organic fraction was dried over $MgSO_4$ and evaporation under reduced pressure afforded pure compounds **20** and **21** as a yellow solid.

*3-Hydroxy-4H-thiopyran-4-thione (**6b**)*

Yield: 3.23g (56%), yellow-orange amorphous solid

^{1}H NMR (400.1 MHz, $CDCl_3$): 7.29 (d, 1H, $J_{2,6}$ = 3.4 Hz, H-2); 7.63 (dd, 1H, $J_{6,5}$ = 9.4 Hz, $J_{6,2}$ = 3.4 Hz, H-6); 8.26 (d, 1H, $J_{5,6}$ = 9.4 Hz, H-5); 9.17 (br s, 1H, OH-3).

^{13}C NMR (100.6 MHz, $CDCl_3$): 106.93 (CH-2); 129.09 (CH-6); 139.59 (CH-5); 159.55 (C-3); 186.11 (C-4).

HRMS: (APCI) m/z calculated for $C_5H_5OS_2$ $[M+H]^+$ 144.97763, found 144.97773

*5-Hydroxy-2-methyl-4H-thiopyran-4-thione (**15b**)*

Compound **15b** was isolated as a mixture with 5-hydroxy-2-methyl-4*H*-pyran-4-thione (**15a**) in ratio 90:10 (GC/MS analysis) and was used for the next step without further purification.

Yield: 3.16g (50%), yellow-orange amorphous solid, contaminated with 6% of **15a**

^{1}H NMR (400.1 MHz, $CDCl_3$): 2.48 (d, 3H, $J_{CH3,3}$ = 0.9 Hz, CH_3-2); 7.15 (s, 1H, H-6); 8.08 (q, 1H, $J_{3,CH3}$ = 0.9 Hz, H-3); 9.07 (bs, 1H, OH-5).

^{13}C NMR (100.6 MHz, $CDCl_3$): 21.98 (CH_3-2); 106.72 (CH-6); 139.26 (CH-3); 143.93 (C-2); 157.61 (C-5); 186.50 (C-4).

MS(EI) m/z (%) = 158.0 (100) [M]+, 129.0 (24), 97.0 (59), 85.0 (19), 69.0 (14), 59.0 (18), 45.0 (43).

*3-Hydroxy-2-methyl-4H-thiopyran-4-thione (**16b**)*

Compound **16b** was isolated as a mixture with 3-hydroxy-2-methyl-4*H*-pyran-4-thione (**16a**) in ratio 85:15 and it was used for the next step without further separation.

Yield: 3.48 g (55%), orange amorphous solid contaminated with 10% of **16a**

NMR and GCMS analyses were in agreement with the literature [5].

*3-Methyl-2H-thiopyrano[3,4-b]furan-2-one (**8**)* [2]

Yield: 423.8 mg (17% after two steps), yellow solid, mp 131-132 °C

^{1}H NMR (500 MHz, Acetone-d_6): 1.91 (s, 3H, CH_3-3); 7.16 (dd, 1H, $J_{7,5}$ = 2.7 Hz, $J_{7,4}$ = 0.5 Hz, H-7); 7.44 (dd, 1H, $J_{4,5}$ = 9.7 Hz, $J_{4,7}$ = 0.5 Hz, H-4); 7.62 (dd, 1H, $J_{5,4}$ = 9.7 Hz, $J_{5,7}$ = 2.7 Hz, H-5).

^{13}C NMR (125.7 MHz, Acetone-d_6): 7.71 (CH_3-3); 102.98 (CH-7); 104.88 (C-3); 119.35 (CH-4); 130.77 (CH-5); 141.26 (C-3a); 147.58 (C-7a); 170.33 (C-2).

HRMS: (ESI+) m/z calculated for $C_8H_7O_2S$ $[M+H]^+$ 167.01613, found 167.01606 .

IR: 3075(m),3061(m), 2912(m), 1733(s), 1628(m), 1600(s), 1364(m), 1295(m), 1172(m), 1162(s), 913(m), 710(m),686(m).

*3,5-Dimethyl-2H-thiopyrano[3,4-b]furan-2-one (**20**)*

Yield: 54.1 mg (2% after two steps), pale-yellow solid, mp 139-140 °C

^{1}H NMR (500 MHz, Acetone-d_6): 1.88 (s, 3H, CH_3-3); 2.42 (d, 3H, $J_{CH3,4}$ = 1.2 Hz, CH_3-5); 7.04 (s, 1H, H-7); 7.20 (q, 1H, $J_{4,CH3}$ = 1.2 Hz, H-4).

^{13}C NMR (125.7 MHz, Acetone-d_6): 7.71 (CH_3-3); 22.33 (CH_3-5); 102.66 (CH-7); 104.39 (C-3); 117.73 (CH-4); 142.57 (C-3a); 143.38 (C-5); 146.73 (C-7a); 170.76 (C-2).

HRMS: (ESI+) m/z calculated for $C_9H_9O_2S$ $[M+H]^+$ 181.03178, found 181.03160.

IR: 3027(m), 2918(m), 2853(m), 1734(s), 1716(s), 1627(m), 1603(m), 1374(m), 1275(m), 1166(m), 913(m), 856(m), 840(m), 747(m), 707(m).

*3,7-Dimethyl-2H-thiopyrano[3,4-b]furan-2-one (**21**)*

Yield: 108.1 mg (4% after two steps), yellow solid, mp 133-135 °C

Alternative procedure: A 1 M solution of LiHMDS (0.22 mL, 0.22 mmol) in 2 mL of anhydrous THF was added dropwise to a stirred solution of **8** (36.3 mg, 0.22 mmol) and MeI (21 µL, 0.33 mmol) in 5 mL of anhydrous THF under inert N_2 atmosphere at −78 °C. Reaction mixture was allowed to warm to −10 °C during 5 h and then stirred for 30 min at laboratory temperature. Reaction mixture was poured into 100 mL of water, 20 mL of 1M HCl was added and crude product was extracted 3 × 30 mL DCM. Combined organic fraction was washed with brine and dried over $MgSO_4$. Column chromatography (SiO_2, DCM) afforded pure product as a yellow solid (23.8 mg, 60%)

^{1}H NMR (500 MHz, Acetone-d_6): 1.89 (s, 3H, CH_3-3); 2.35 (s, 3H, CH_3-7); 7.35 (d, 1H, $J_{4,5}$ = 9.7 Hz, H-4); 7.53 (d, 1H, $J_{5,4}$ = 9.7 Hz, H-5).

^{13}C NMR (125.7 MHz, Acetone-d_6): 7.89 (CH_3-3); 15.09 (CH_3-7); 104.89 (C-3); 115.18 (C-7); 118.84 (CH-4); 130.69 (CH-5); 141.71 (C-3a); 144.30 (C-7a); 170.36 (C-2).

HRMS: (APCI) m/z calculated for $C_9H_9O_2S$ [M+H]$^+$ 181.03178, found 181.03168.
IR: 3039(m), 3014(m), 2916(m), 2853(m), 1717(s), 1683(m), 1601(m), 1342m), 1266(m), 1128(m), 695(m).

*4,7-Dihydroxy-4,5,7,7a-tetrahydro-2H-thiopyrano[3,4-b]furan-2-one (**23**)* [6]

Ethyl (*E*)-2-(5-((acetylthio)methyl)-2,2-dimethyldihydrofuro[2,3-*d*][1,3]dioxol-6(5*H*)-ylidene) acetate (**22**, 4.83 g, 15.3 mmol) was dissolved in a mixture MeOH/H_2O 1.5/1 (700 mL) and argon was bubbled through for 25 min. An aqueous solution of NaOH (10 M, 50 mL) was prepared and argon was bubbled through for 1 h. A solution of NaOH (2.9 mL, 10 M) was added to the reaction mixture under argon atmosphere and the reaction was stirred for 9 min. The reaction mixture was made neutral by addition of Amberlyst 15 (in H^+ cycle). The suspension was filtered off, washed with 2 × 50 mL MeOH and the filtrate was evaporated under reduced pressure. The solid residue was refluxed for 2 h in aqueous acetic acid (70%, 160 mL) under argon atmosphere. The reaction mixture was evaporated under reduced pressure and the solid residue was purified by column chromatography (SiO_2, 50% EtOAc in petroleum ether 40-60) to obtain the title compound **23** (0.96 g, 33%).
NMR and GCMS analyses were in agreement with the published data [6].

*Ethyl (2-oxo-4,5-dihydro-2H-thiopyrano[3,4-b]furan-4-yl) carbonate (**25**)*

Ethyl chloroformate (2.2 mL, 23.12 mmol) was added to a stirred solution of **23** (1.088 g, 5.78 mmol) in dry pyridine (23.5 mL) at 0 °C under argon atmosphere. The reaction mixture was stirred 1 h at room temperature. The reaction mixture was evaporated to dryness under reduced pressure and the residue was partitioned between water and DCM. The organic layer was dried over $MgSO_4$ and evaporated to dryness under reduced pressure to obtain a mixture of title compound **25** and dicarbonylated compound **24**. The residue was dissolved in DCM (50 mL) and triethylamine (3 mL) was added. The reaction was stirred overnight. The reaction mixture was washed with a saturated solution of $NaHCO_3$ and the aqueous layer was extracted with DCM (3 × 20 mL). The combined organic layer was washed with 10% citric acid, brine, dried over $MgSO_4$ and evaporated to dryness under reduced pressure. The residue was purified by column chromatography (SiO_2, 15% EtOAc in petroleum ether 40–60) to obtain the title compound **25** (0.626 g, 44% over two steps).

^{1}H NMR (401 MHz, $CDCl_3$): 1.35 (t, 3H, $J_{CH3,CH2}$ = 7.1 Hz, CH_3CH_2O); 3.18 – 3.27 (m, 2H, H-5); 4.28 (q, 2H, $J_{CH2,CH3}$ = 7.1 Hz, CH_3CH_2O); 5.84 (ddd, 1H, $J_{4,5}$ = 7.9 and 6.9 Hz, $J_{4,3}$ = 1.6 Hz, H-4); 6.10 (t, 1H, $J_{3,4}$ = $J_{3,7}$ = 1.7 Hz, H-3); 6.46 (d, 1H, $J_{7,3}$ = 1.8 Hz, H-7).

^{13}C NMR (100.8 MHz, $CDCl_3$): 14.13 (CH_3CH_2OCO); 31.49 (CH_2-5); 65.20 (CH_3CH_2OCO); 69.45 (CH-4); 108.89 (CH-7); 112.86 (CH-3); 143.78 (C-7a); 148.71 (C-3a); 153.87 (CH_3CH_2OCO); 167.49 (C-2).

HRMS (APCI) m/z calculated for $C_{10}H_{11}O_5S$ $[M+H]^+$ 243.0321, found 243.0321.

*2H-Thiopyrano[3,4-b]furan-2-one (**26**)*

Ethyl (2-oxo-4,5-dihydro-2*H*-thiopyrano[3,4-*b*]furan-4-yl) carbonate (**25**) (0.316 g, 1.304 mmol) was dissolved in anhydrous toluene (10 mL) and bis *N,O*-trimethylsilyl acetamide (0.2 mL) was added under an inert atmosphere of argon. The reaction mixture was stirred for 10 min. Tetrakis(triphenylphosphine)palladium (0.186 g, 0.161 mmol) was added and the reaction was stirred overnight under argon atmosphere at 60 °C. The solvent was removed under vacuum and the residue was purified by column chromatography (30% EtOAc in petroleum ether 40–60) to give the title compound **26** (0.18 g, 74%), beige amorphous solid.

^{1}H NMR (401 MHz, $CDCl_3$): 5.64 (d, 1H, $J_{3,7}$ = 1.5 Hz, H-3); 6.98 (dt, 1H, $J_{7,5}$ = 2.4 Hz, $J_{7,4}$ = $J_{7,3}$ = 1.4 Hz, H-7); 7.33 - 7.40 (m, 2H, H-4,5).

^{13}C NMR (100.8 MHz, $CDCl_3$): 95.73 (CH-3); 103.85 (CH-7); 120.01 (CH-4); 130.48 (CH-5); 145.47 and 147.91 (C-3a,7a); 169.54 (C-2).

HRMS (APCI) m/z calculated for $C_7H_5O_2S$ $[M+H]^+$ 153.0006, found 153.0005.

IR: 3061(m), 1760(m), 1716(s), 1620(m), 1607(m), 1572(s), 1407(m), 1361(m), 1258(m), 1172(m), 1162(s), 913(m), 710(m),686(m).

Synthesis of KAR analogues with sulfur in positions 2 and 6

General procedure

The corresponding thiokarrikin (**8**, **20**, **21** and **26**) (0.2 mmol, 1 equiv), Lawesson´s reagent (80.9 mg, 0.2 mmol, 1 equiv) and HMDO (55.0µM, 0.26 mmol, 1.3 equiv) were suspended in anhydrous toluene (5 mL), heated and stirred in a microwave reactor (300 W) for 60 min at 120 °C. After cooling, the solvent was removed under vacuum and column chromatography (SiO_2, DCM) afforded the title compound.

*3-Methyl-2H-thiopyrano[3,4-b]furan-2-thione (**27**)*

Yield: 22.2 mg (61%), orange-red solid, mp 170-172 °C

^{1}H NMR (500 MHz, Acetone-d$_6$): 2.10 (s, 3H, CH$_3$-3); 7.67 (d, 1H, $J_{4,5}$ = 9.6 Hz, H-4); 7.76 (d, 1H, $J_{7,5}$ = 2.6 Hz, H-7); 7.93 (dd, 1H, $J_{5,4}$ = 9.6 Hz, $J_{5,7}$ = 2.6 Hz, H-5).

^{13}C NMR (125.7 MHz, Acetone-d$_6$): 9.94 (CH$_3$-3); 107.80 (CH-7); 121.05 (CH-4); 122.78 (C-3); 132.79 (CH-5); 140.33 (C-3a); 154.07 (C-7a); 200.56 (C-2).

HRMS: (APCI) m/z calculated for $C_8H_7OS_2$ $[M+H]^+$ 182.99328, found 182.99317.

IR: 3020(m), 2911(m), 2853(m), 1614(m), 1597(m), 1573(m), 1445(m), 1378(m), 1368(s), 1342(m), 1326(m), 1173(m), 1153(m), 1045(m), 894(m), 699(m).

*3,5-Dimethyl-2H-thiopyrano[3,4-b]furan-2-thione (**28**)*

Yield: 31.4 mg (80%), orange solid, mp 187-188 °C

^{1}H NMR (500 MHz, Acetone-d$_6$): 2.07 (s, 3H, CH$_3$-3); 2.54 (q, 3H, $J_{CH3,4}$ = 1.2 Hz, CH$_3$-5); 7.43 (q, 1H, $J_{4,CH3}$ = 1.2 Hz, H-4); 7.63 (s, 1H, H-7).

^{13}C NMR (125.7 MHz, Acetone-d$_6$): 9.93 (CH$_3$-3); 22.32 (CH$_3$-5); 107.21 (CH-7); 119.51 (CH-4); 122.18 (C-3); 141.75 (C-3a); 146.03 (C-5); 153.18 (C-7a); 201.10 (C-2).

HRMS: (ESI+) m/z calculated for $C_9H_9OS_2$ $[M+H]^+$ 197.00893, found 197.00886.

IR: 3026(m), *2993(m), 2925(m),* 1611(m), 1600(m), 1562(m), 1495(m), 1377(m), 1337(m), 1112(m), 1042(m), 816(s), 700(m).

*3,7-Dimethyl-2H-thiopyrano[3,4-b]furan-2-thione (**29**)*

Yield: 25.9 mg (66%), orange-red solid, mp 171-172 °C

^{1}H NMR (500 MHz, Acetone-d$_6$): 2.09 (s, 3H, CH$_3$-3); 2.55 (s, 3H, CH$_3$-7); 7.59 (d, 1H, $J_{4,5}$ = 9.5 Hz, H-4); 7.85 (d, 1H, $J_{5,4}$ = 9.5 Hz, H-5).

^{13}C NMR (125.7 MHz, Acetone-d$_6$): 10.16 (CH$_3$-3); 15.72 (CH$_3$-7); 120.55 (CH-4); 121.38 (C-7); 122.86 (C-3); 132.72 (CH-5); 140.71 (C-3a); 151.37 (C-7a); 200.02 (C-2).

HRMS: HRMS: (ESI+) m/z calculated for $C_9H_9OS_2$ $[M+H]^+$ 197.00893, found 197.00889.

IR: 3064(m),3014(m), 2925(m), 2851(m), 1617(m), 1578(m), 1445(m), 1378(m), 1368(s), 1342(m), 1326(m), 1173(m), 1153(m), 1045(m), 894(m), 699(m).

*2H-Thiopyrano[3,4-b]furan-2-thione (**30**)*

Yield: 14.1 mg (42%), dark red solid, mp 185-187 °C

^{1}H NMR (401 MHz, $CDCl_3$): 6.54 (d, 1H, $J_{3,7}$ = 1.4 Hz, H-3); 7.38 (dt, 1H, $J_{7,5}$ = 2.2 Hz, $J_{7,4}$ = $J_{7,3}$ = 1.2 Hz, H-7); 7.48 - 7.55 (m, 2H, H-4,5).

^{13}C NMR (100.8 MHz, $CDCl_3$): 106.69 (CH-7); 115.75 (CH-3); 120.94 (CH-4); 131.64 (CH-5); 143.81 (C-3a); 154.71 (C-7a); 199.39 (C-2).

HRMS: (APCI) m/z calculated for $C_7H_5OS_2$ $[M+H]^+$ 168.97763, found 168.97764.

IR: 3057(m), 2923(m), 2853(m), 1620(m), 1580(m), 1448(m), 1352(s), 1342(m), 1171(m), 1147(m), 1087(m), 874(m), 699(m).

Copies of NMR Spectra

3-Methyl-2*H*-furo[2,3-*c*]pyran-2-thione (**9**)

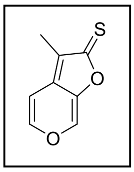

^{1}H NMR (acetone-d_6)

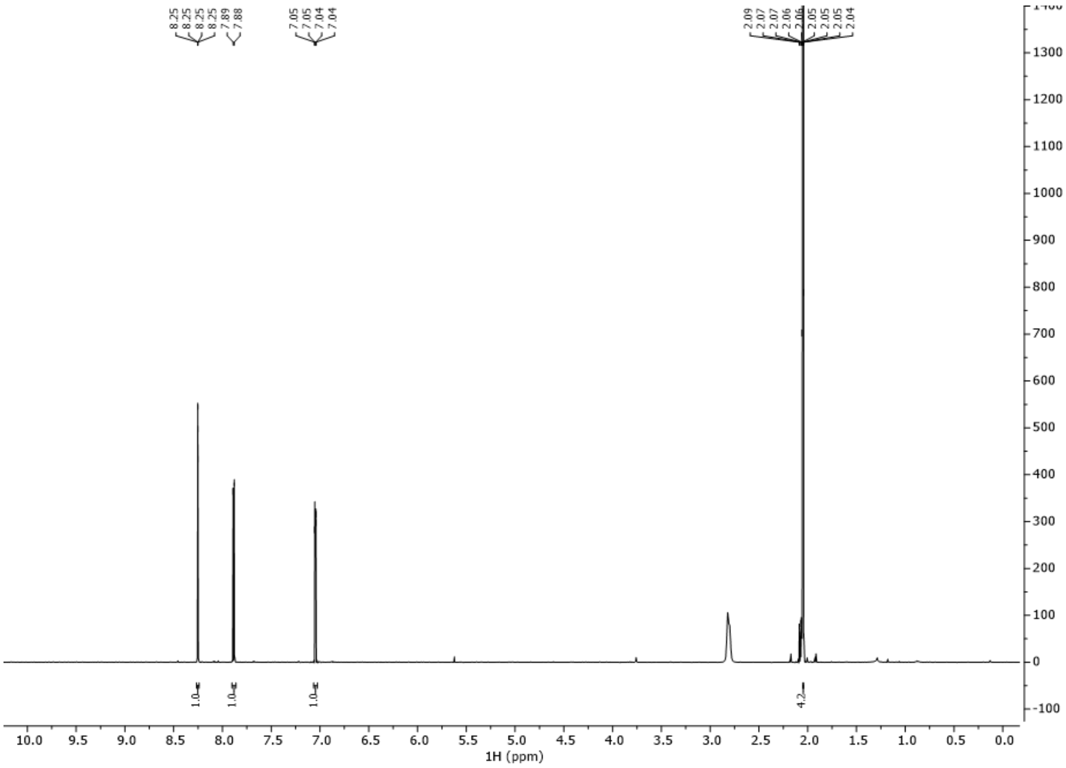

APT (acetone-d_6)

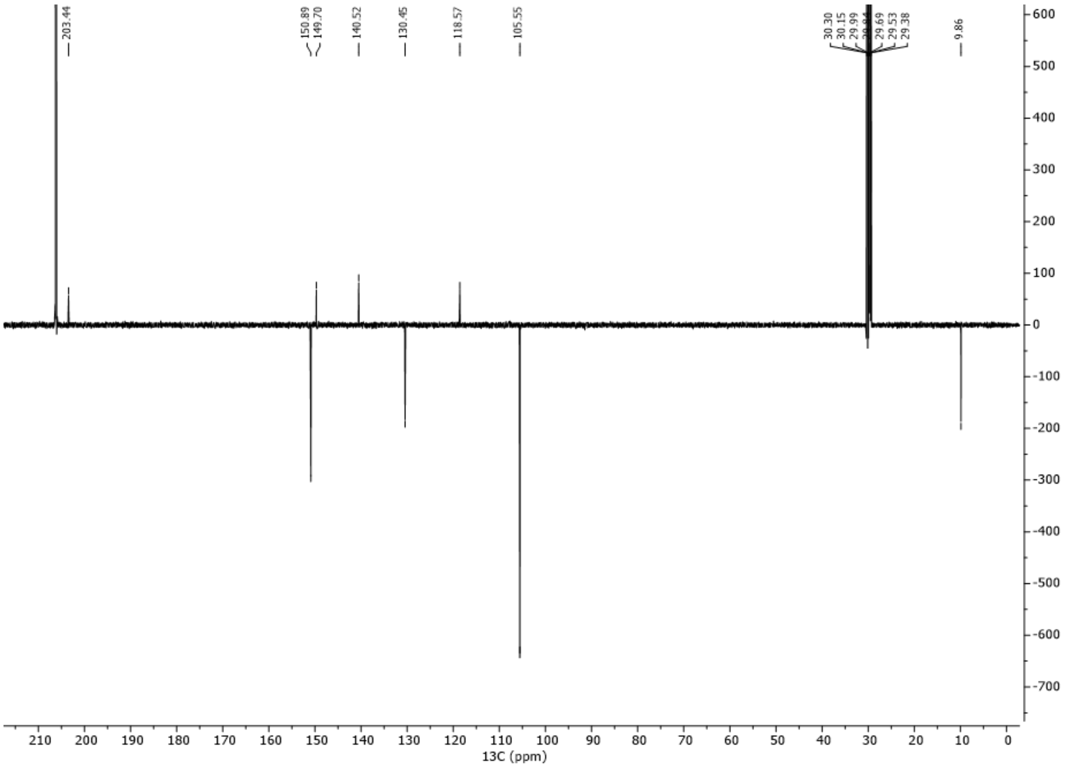

2*H*-Furo[2,3-*c*]pyran-2-thione (**10**)

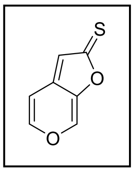

^{1}H NMR (acetone-d_6)

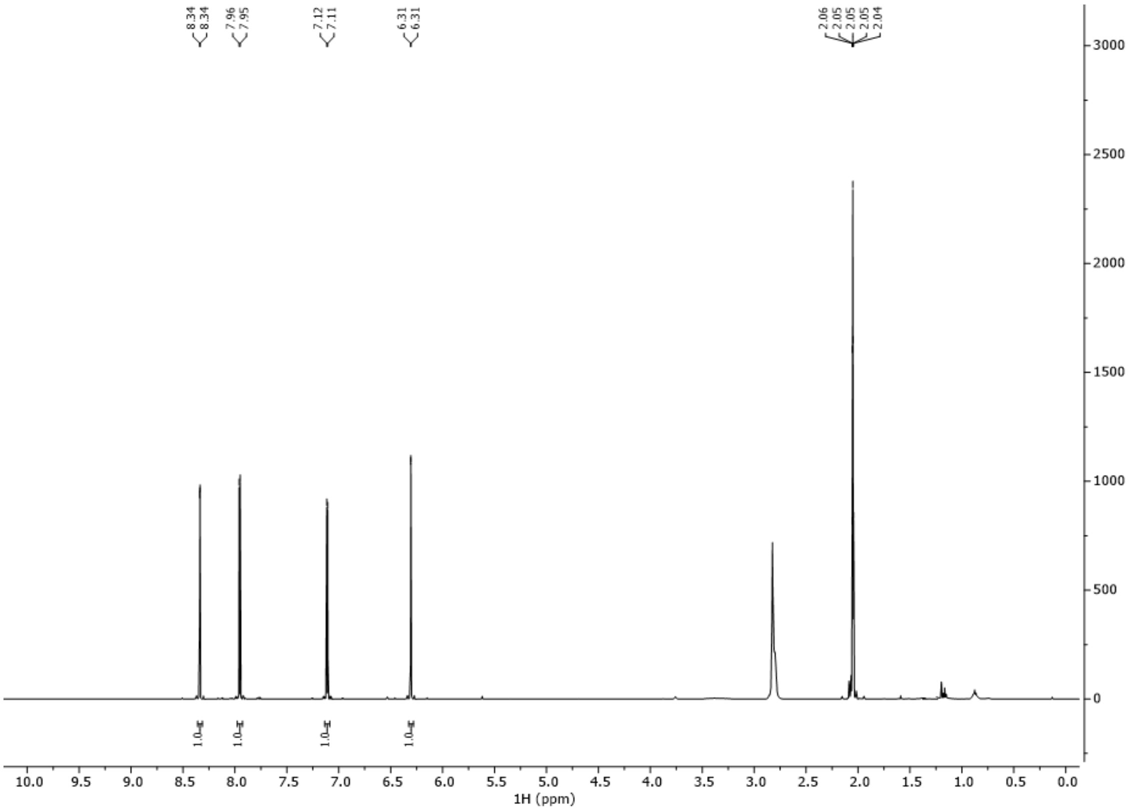

APT (acetone-d_6)

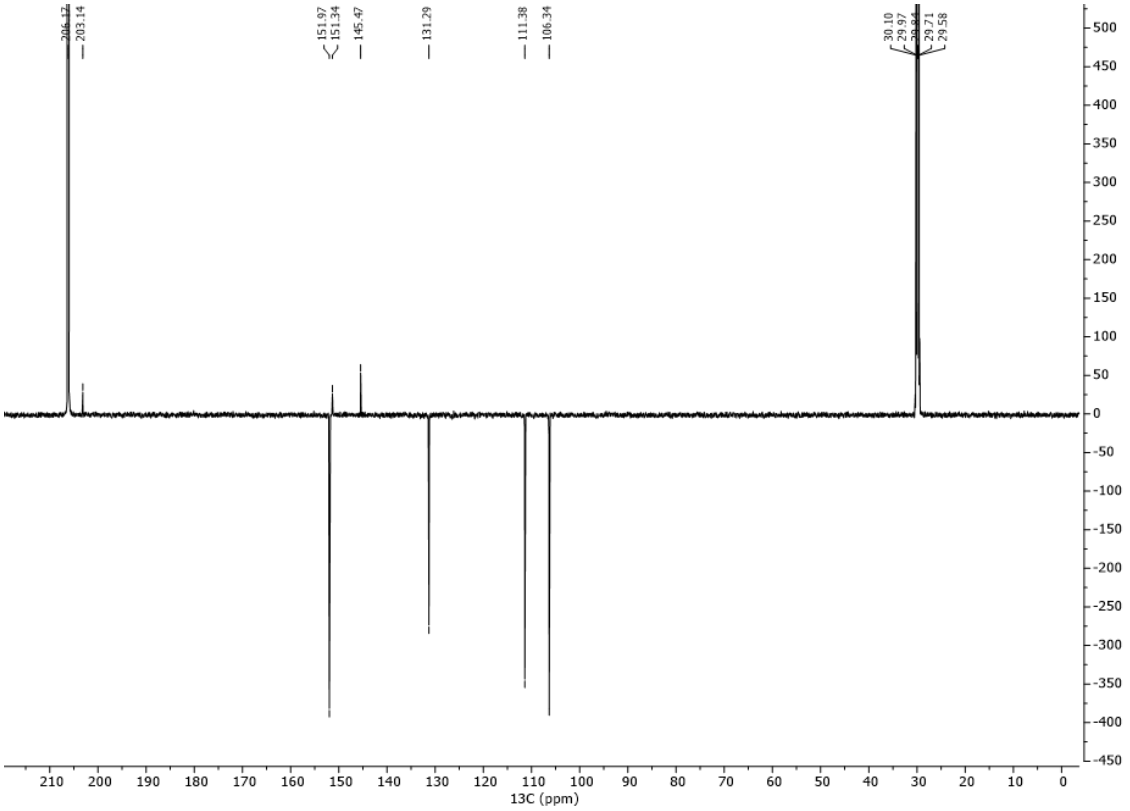

3,5-Dimethyl-2*H*-furo[2,3-*c*]pyran-2-thione (**11**)

^{1}H NMR (acetone-d_6)

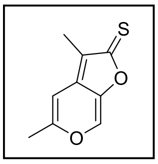

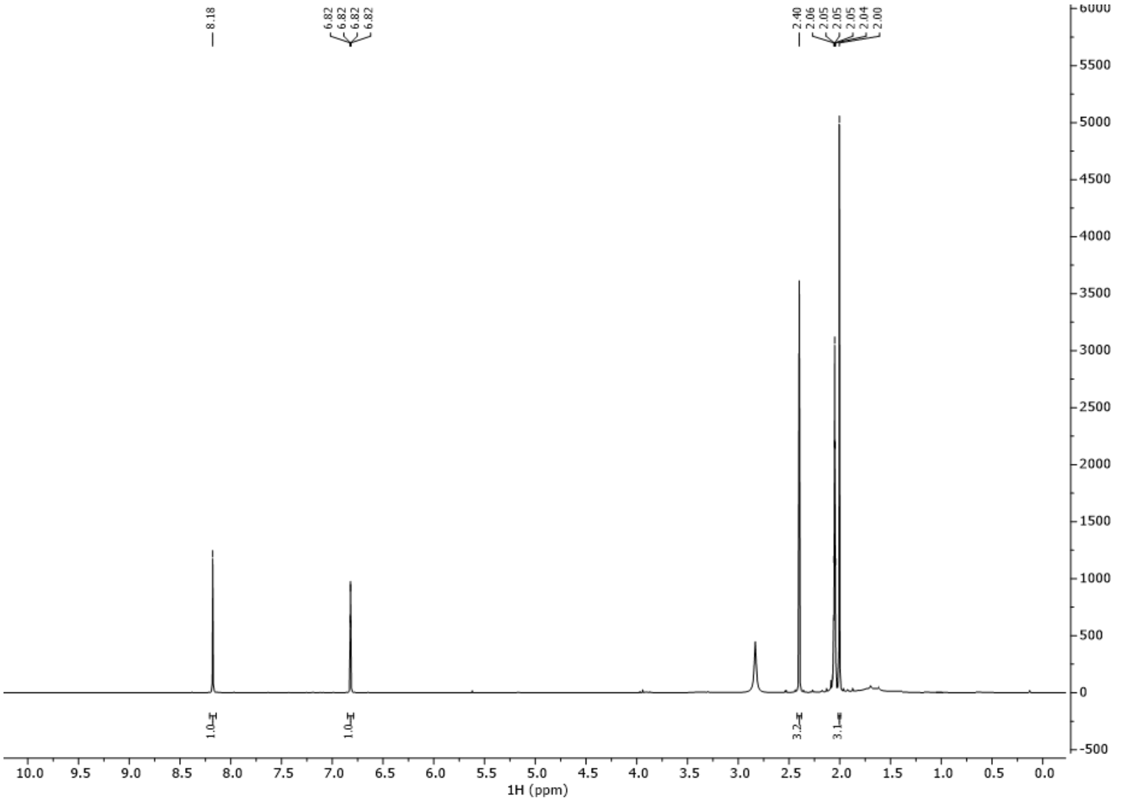

APT (acetone-d_6)

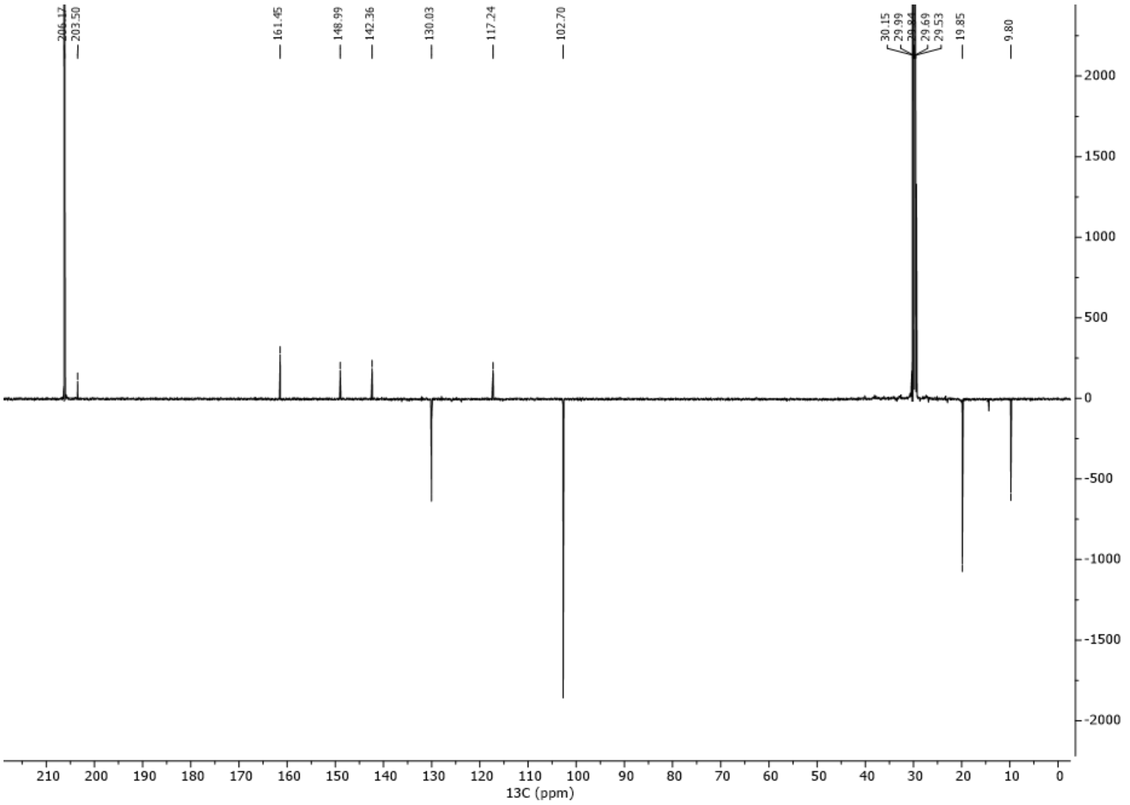

3,7-Dimethyl-2*H*-furo[2,3-*c*]pyran-2-thione (**12**)

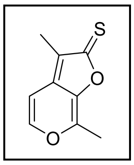

^{1}H NMR (acetone-d_6)

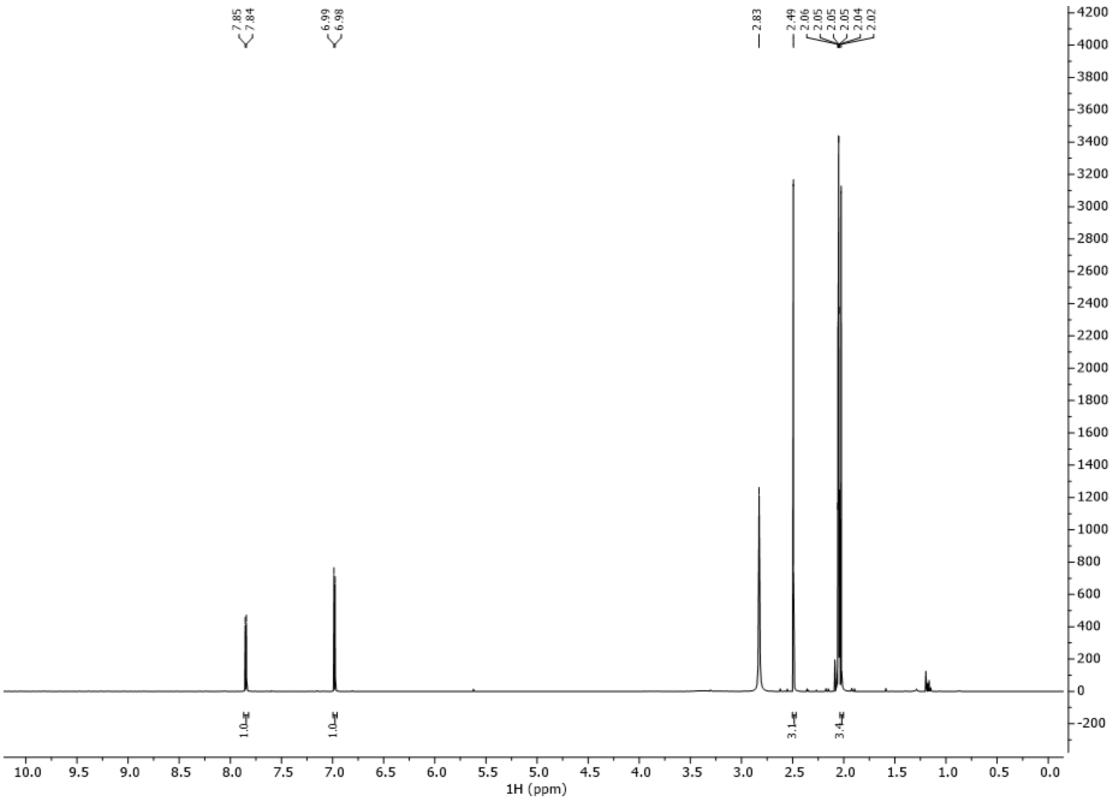

APT (acetone-d_6)

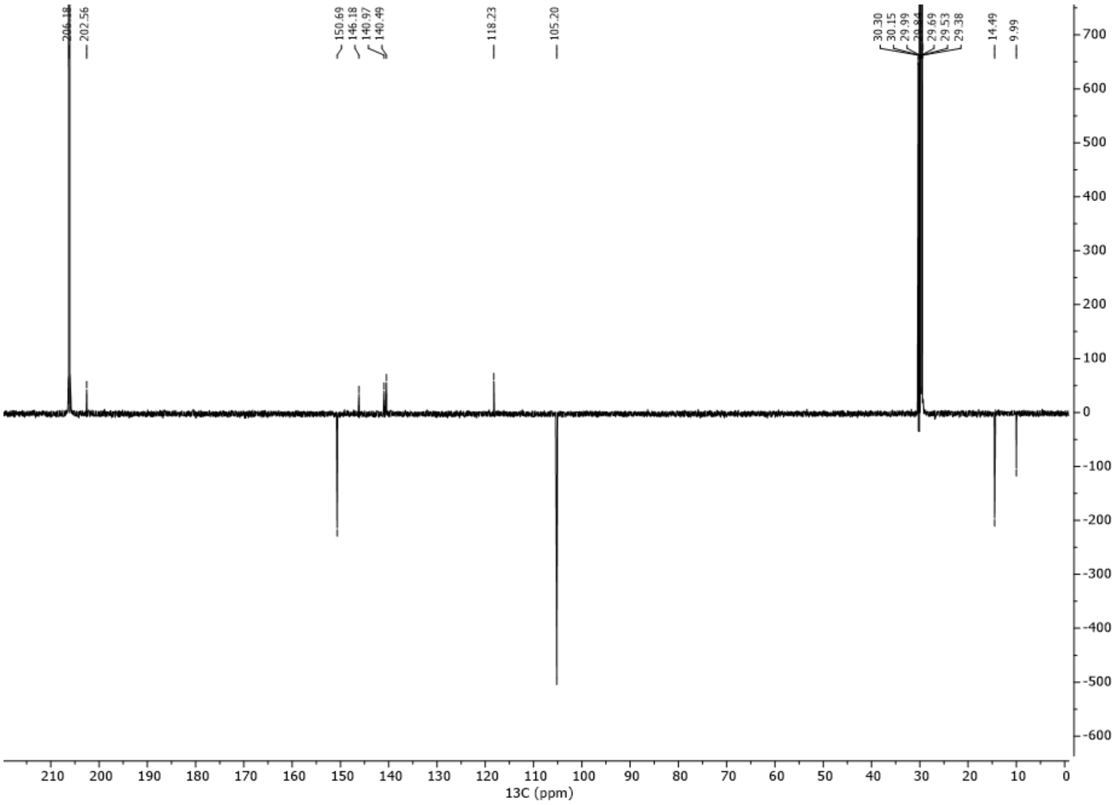

3-Hydroxy-4*H*-thiopyran-4-thione (**6b**)

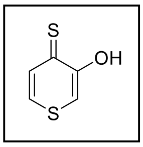

^{1}H NMR ($CDCl_3$)

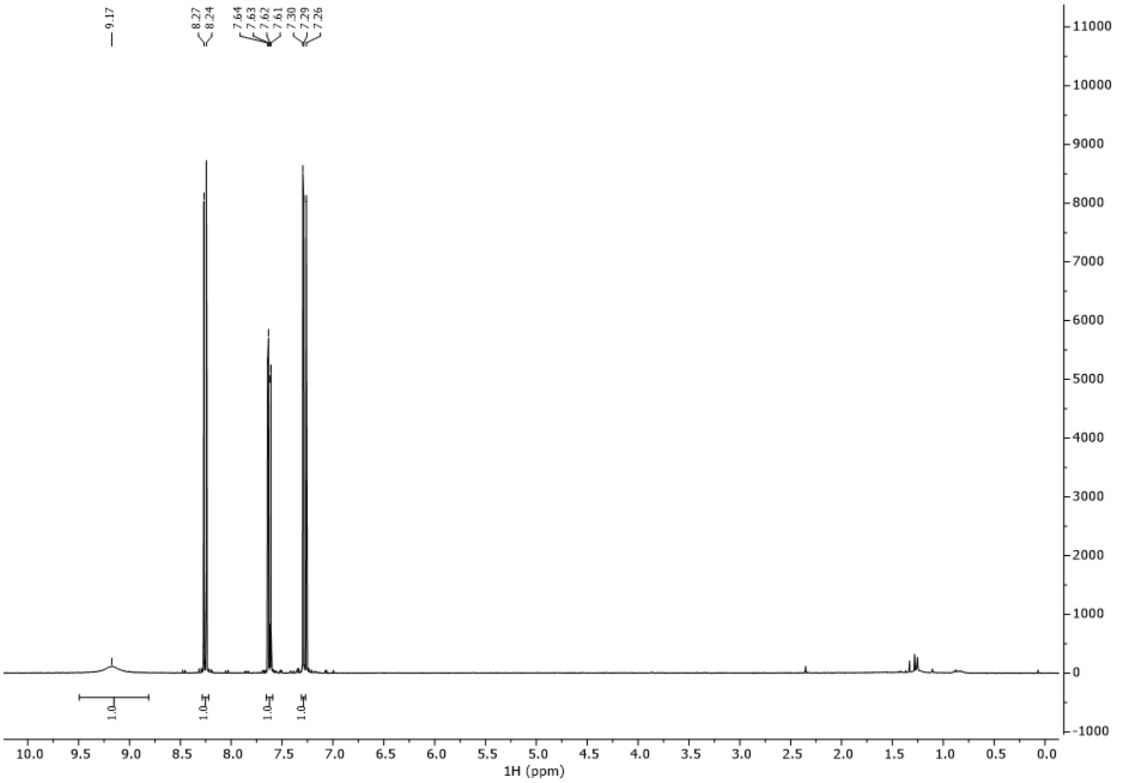

APT ($CDCl_3$)

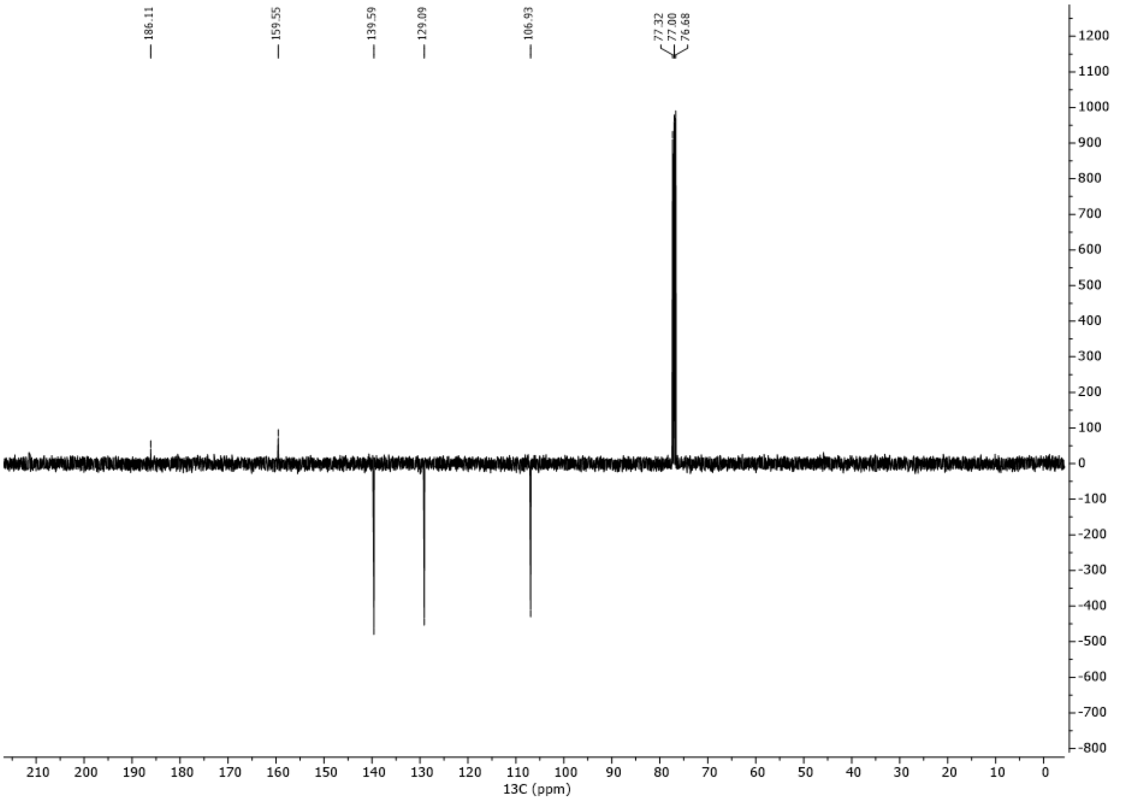

5-Hydroxy-2-methyl-4*H*-thiopyran-4-thione (**15b**)

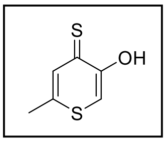

^{1}H NMR ($CDCl_3$)

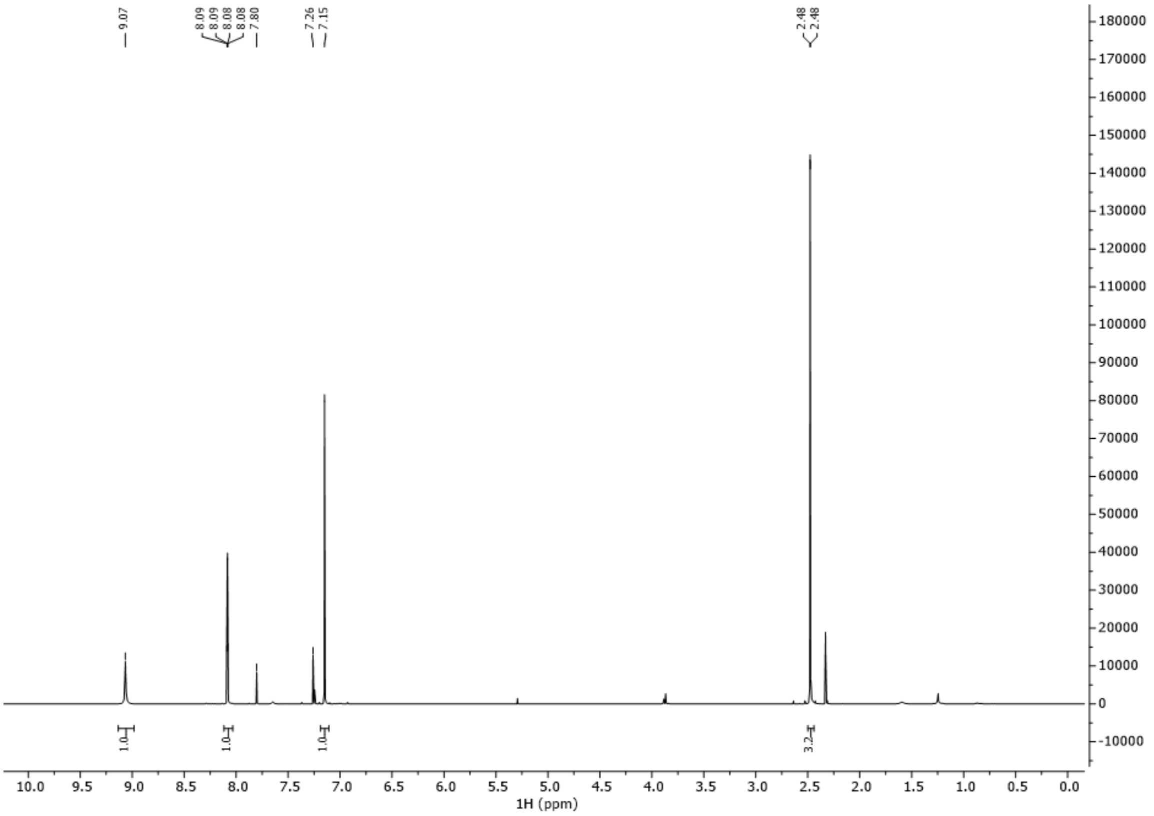

APT ($CDCl_3$)

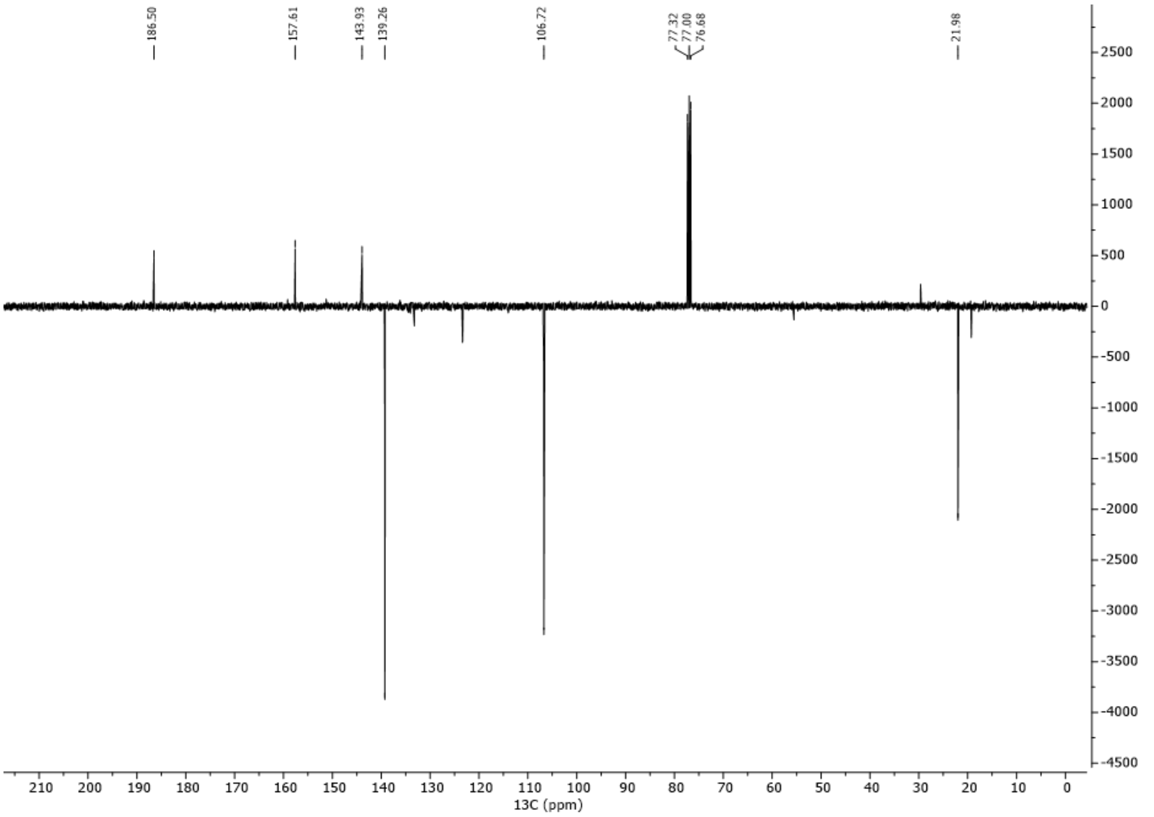

3-Methyl-2*H*-thiopyrano[3,4-*b*]furan-2-one (**8**)

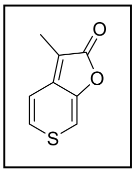

^{1}H NMR (acetone-d_6)

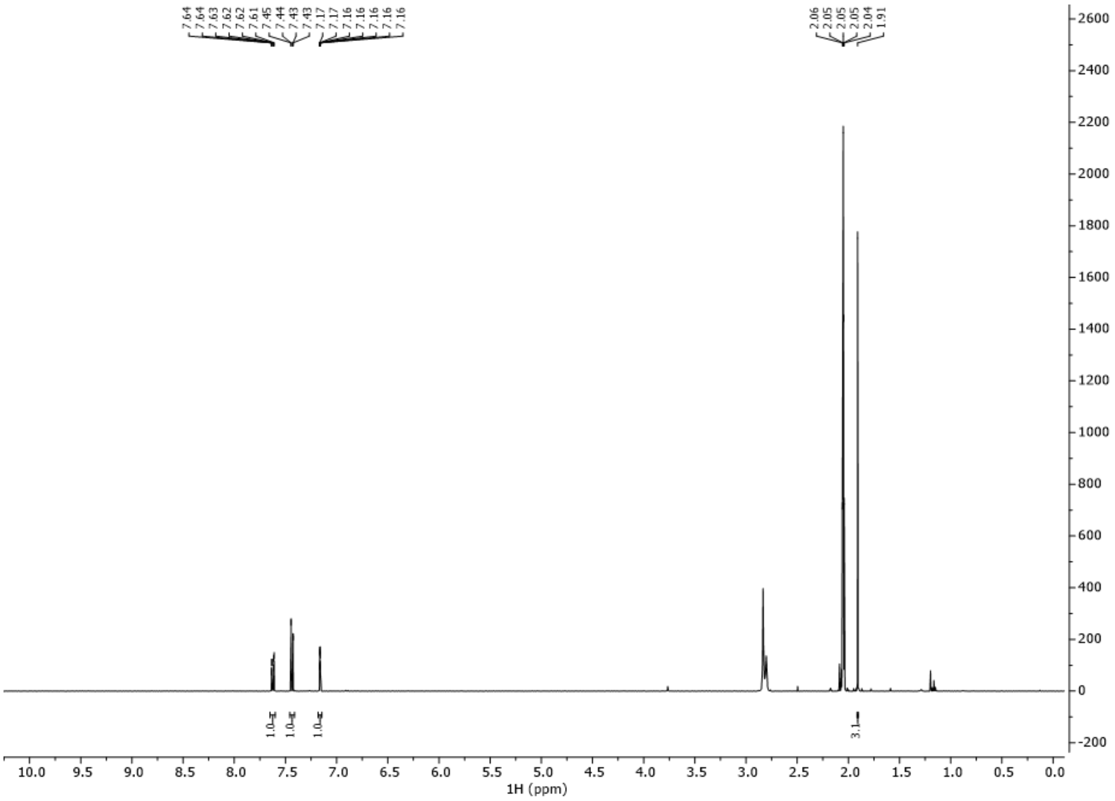

APT (acetone-d_6)

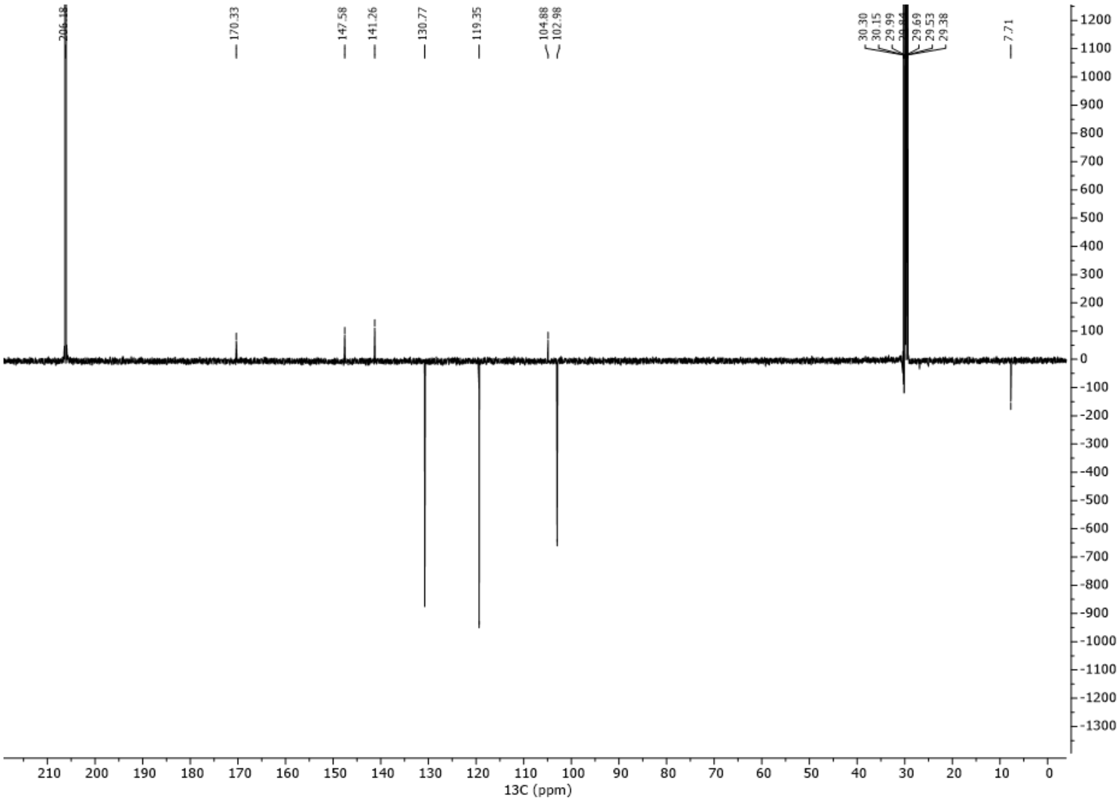

3,5-Dimethyl-2*H*-thiopyrano[3,4-*b*]furan-2-one (**20**)

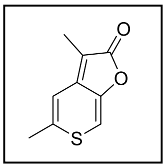

^{1}H NMR (acetone-d_6)

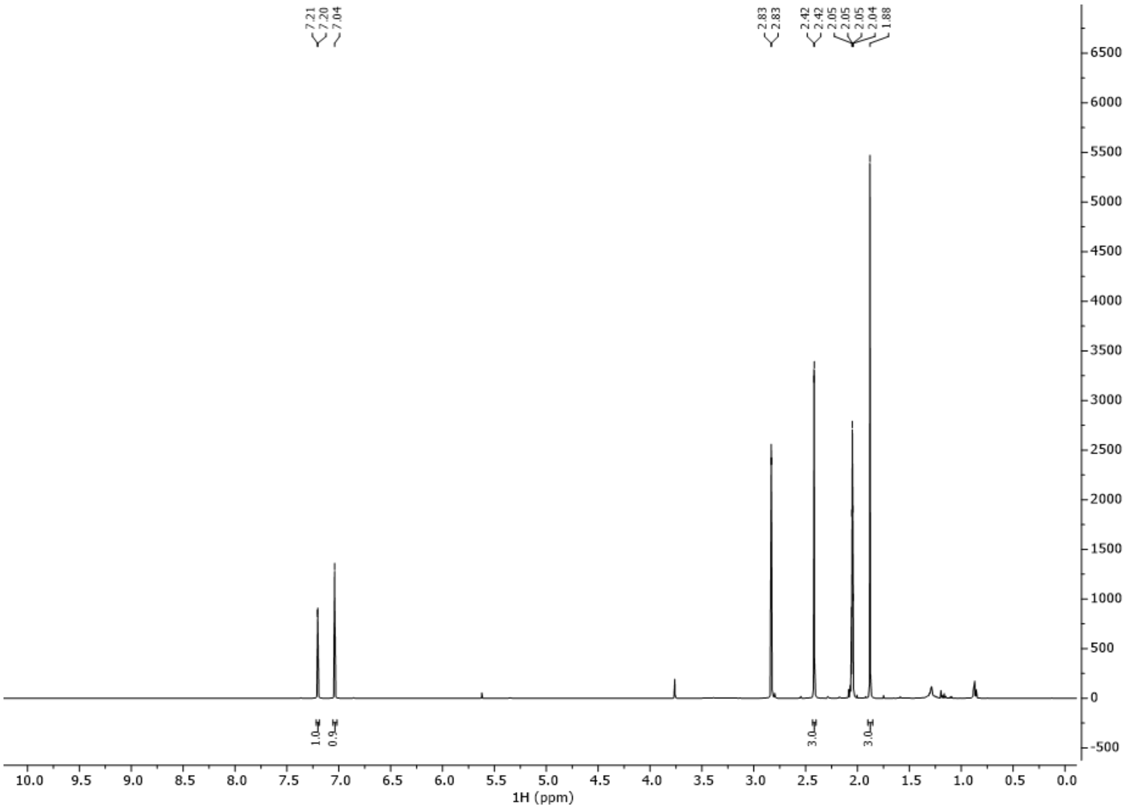

APT (acetone-d_6)

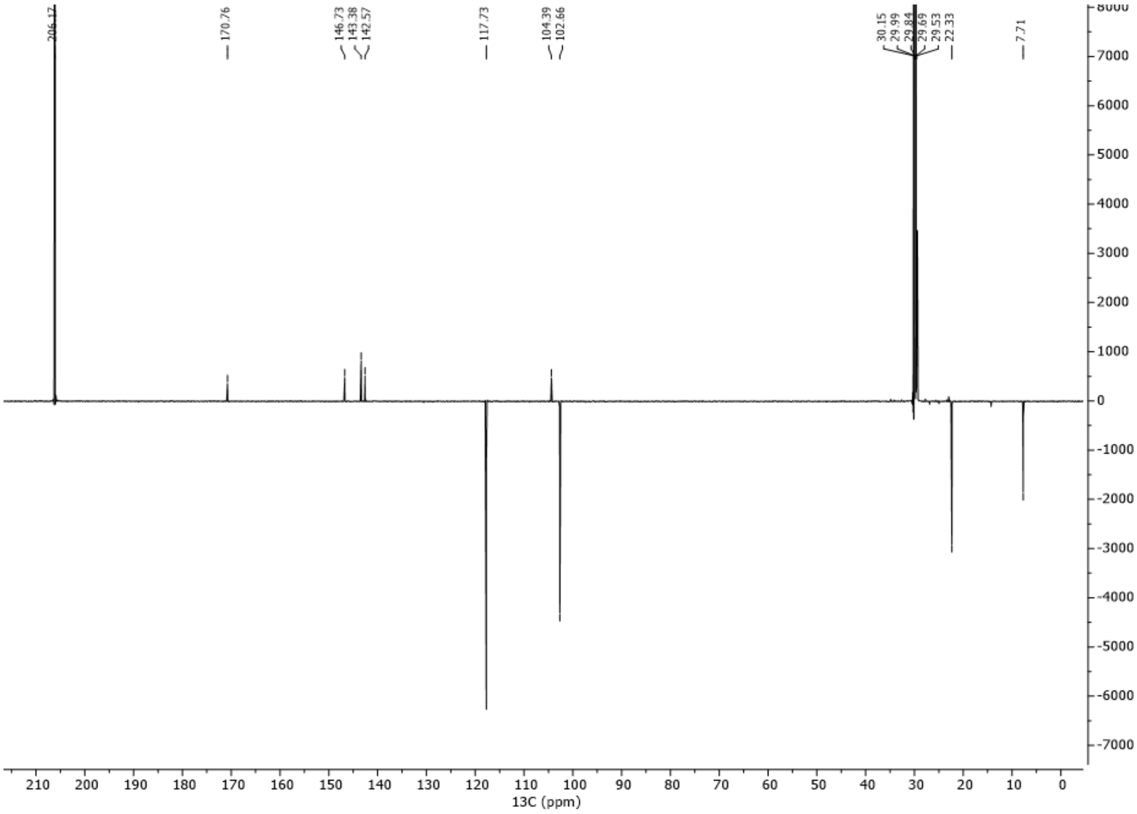

3,7-Dimethyl-2*H*-thiopyrano[3,4-*b*]furan-2-one (**21**)

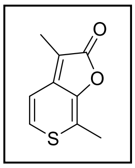

^{1}H NMR (acetone-d_6)

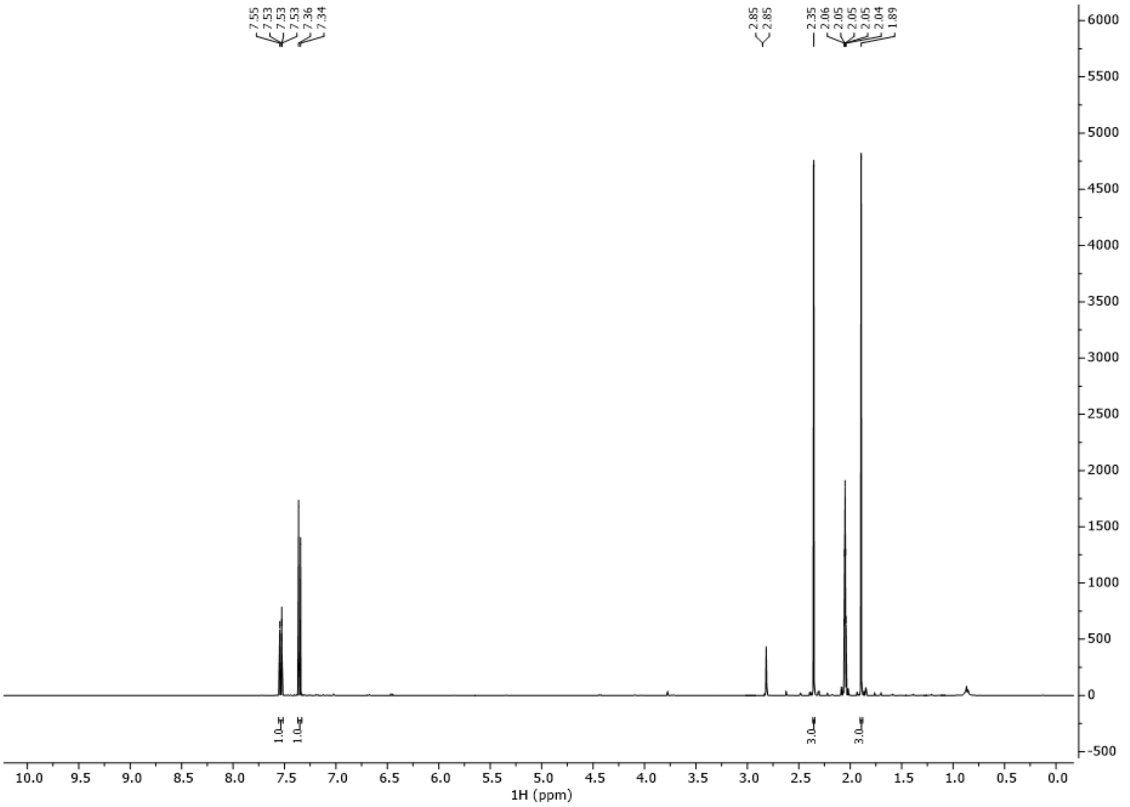

APT (acetone-d_6)

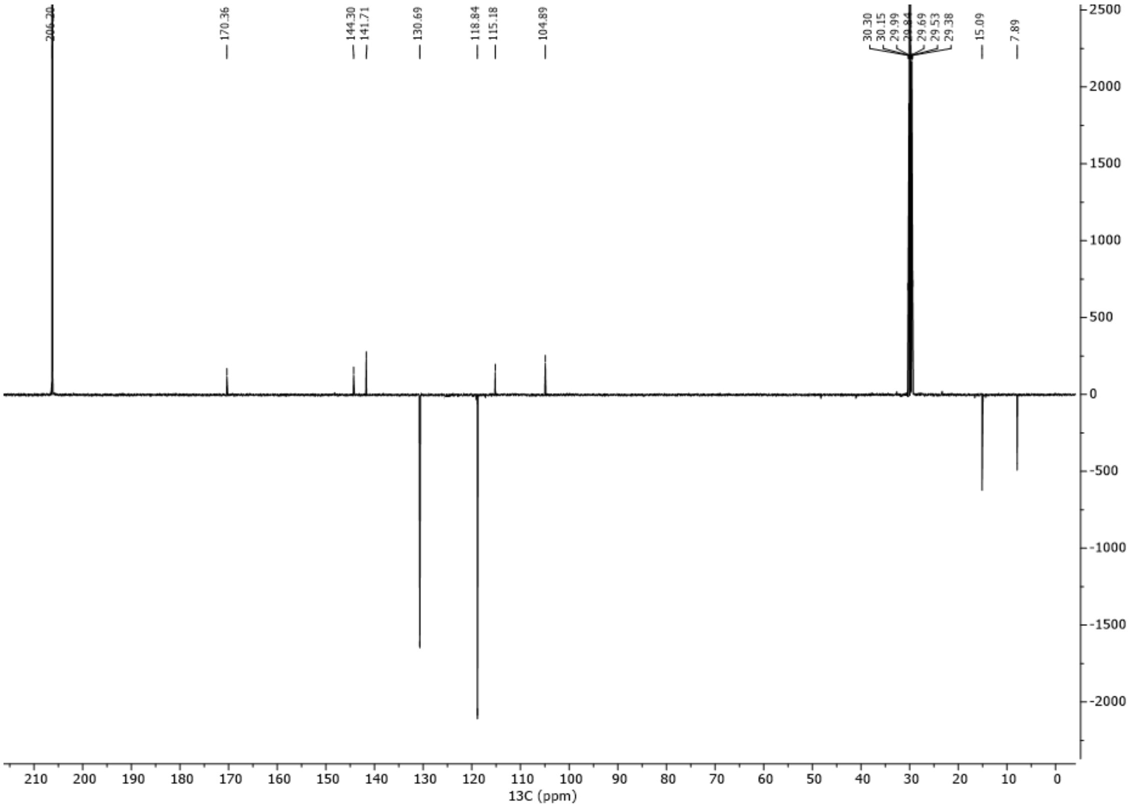

Ethyl (2-oxo-4,5-dihydro-2*H*-thiopyrano[3,4-*b*]furan-4-yl) carbonate (**25**)

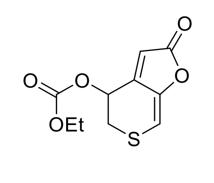

^{1}H NMR ($CDCl_3$)

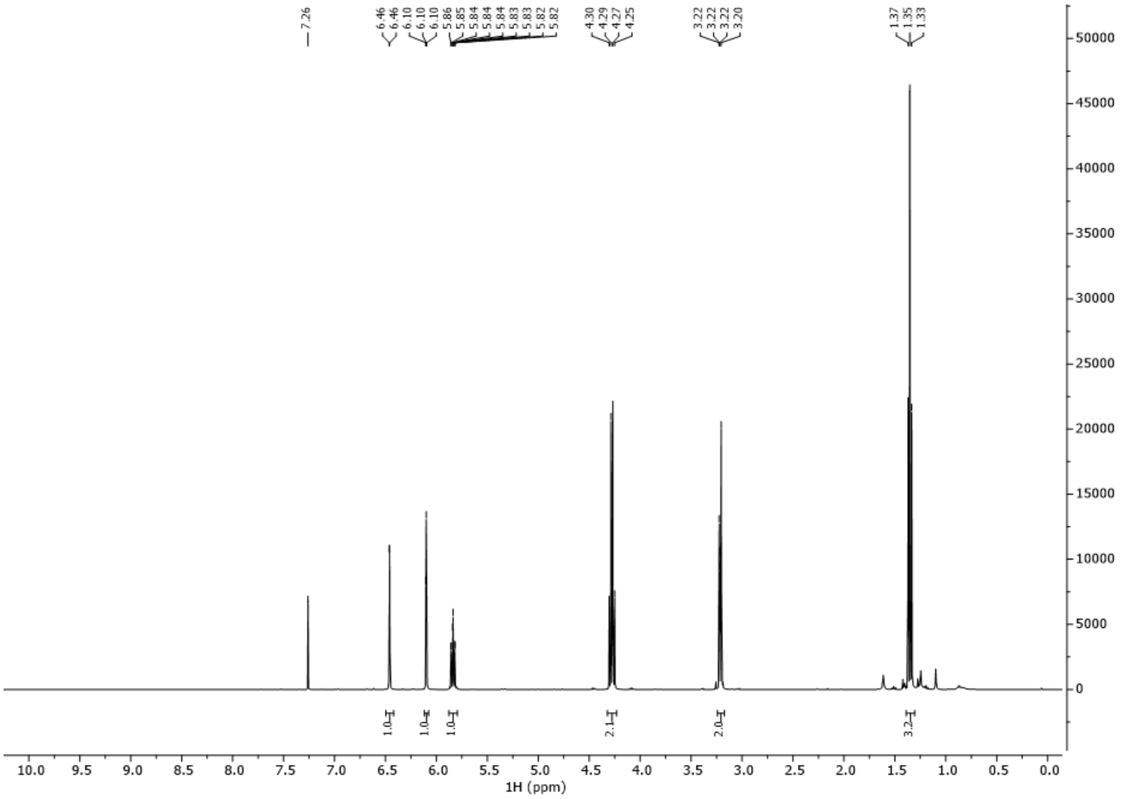

APT ($CDCl_3$)

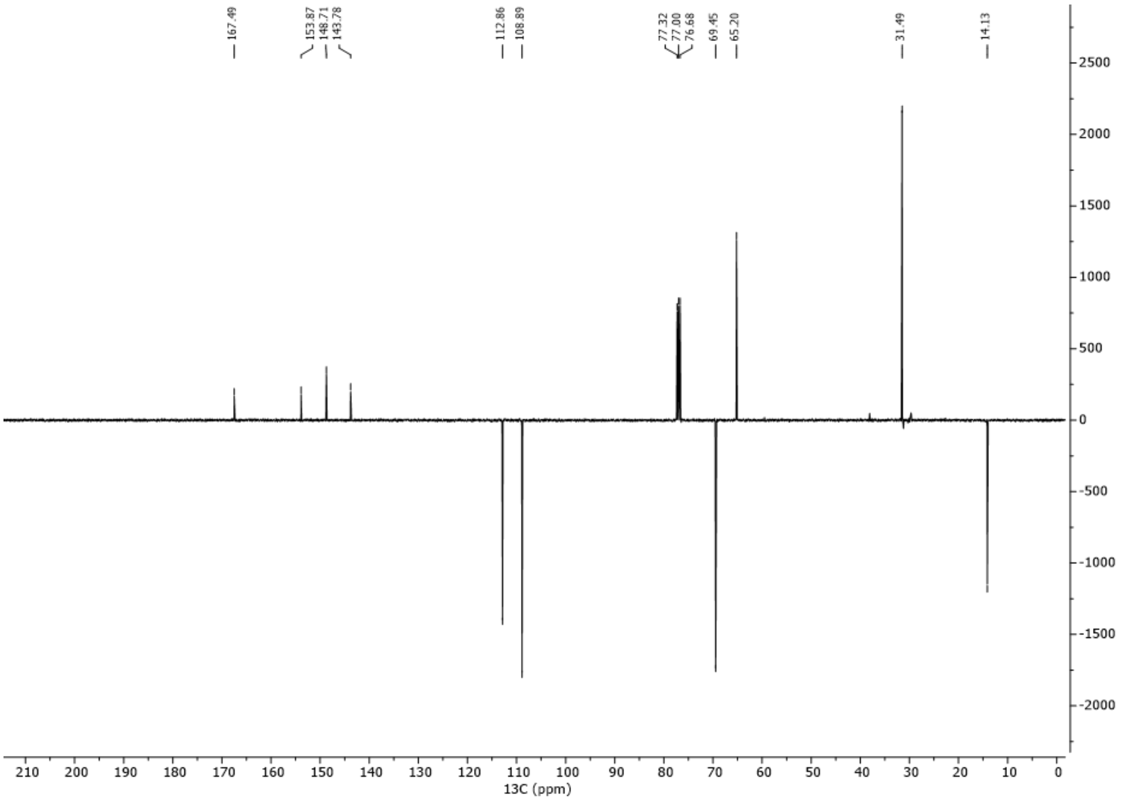

2*H*-Thiopyrano[3,4-*b*]furan-2-one (**26**)

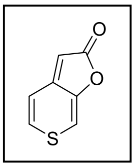

^{1}H NMR ($CDCl_3$)

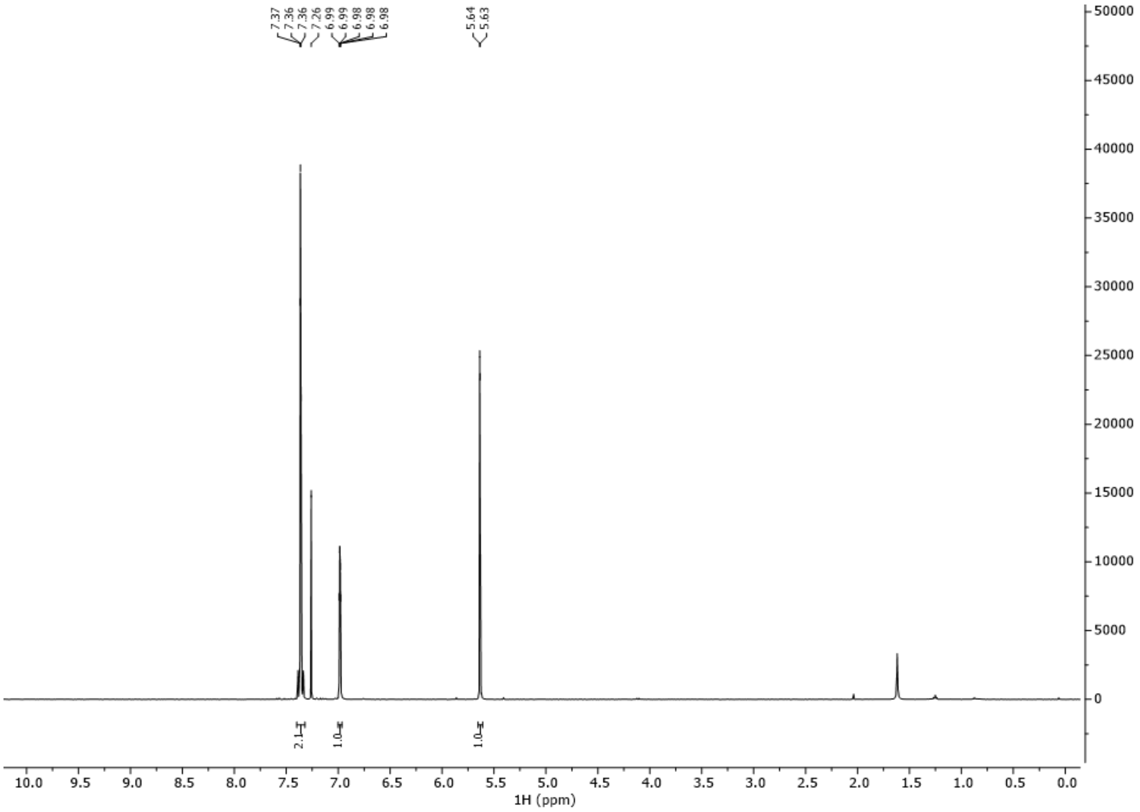

APT ($CDCl_3$)

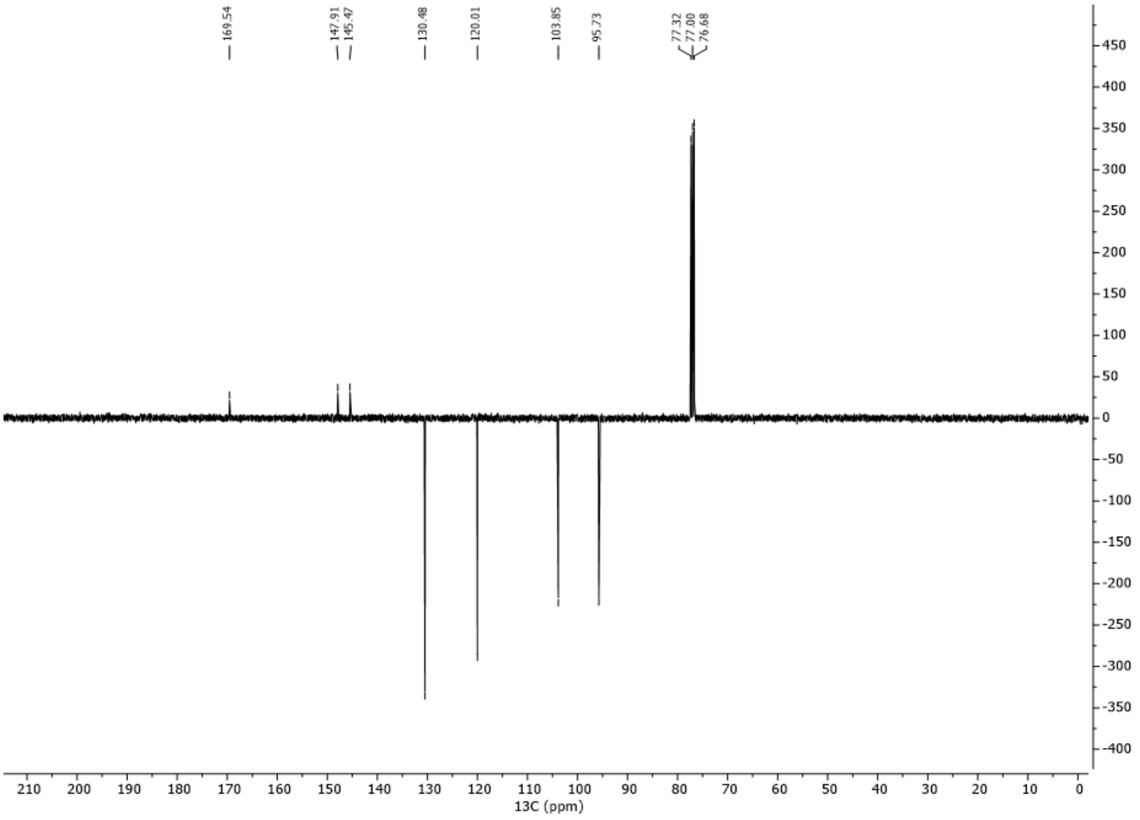

3-Methyl-2*H*-thiopyrano[3,4-*b*]furan-2-thione (**27**)

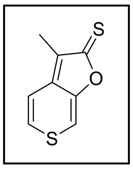

^{1}H NMR (acetone-d_6)

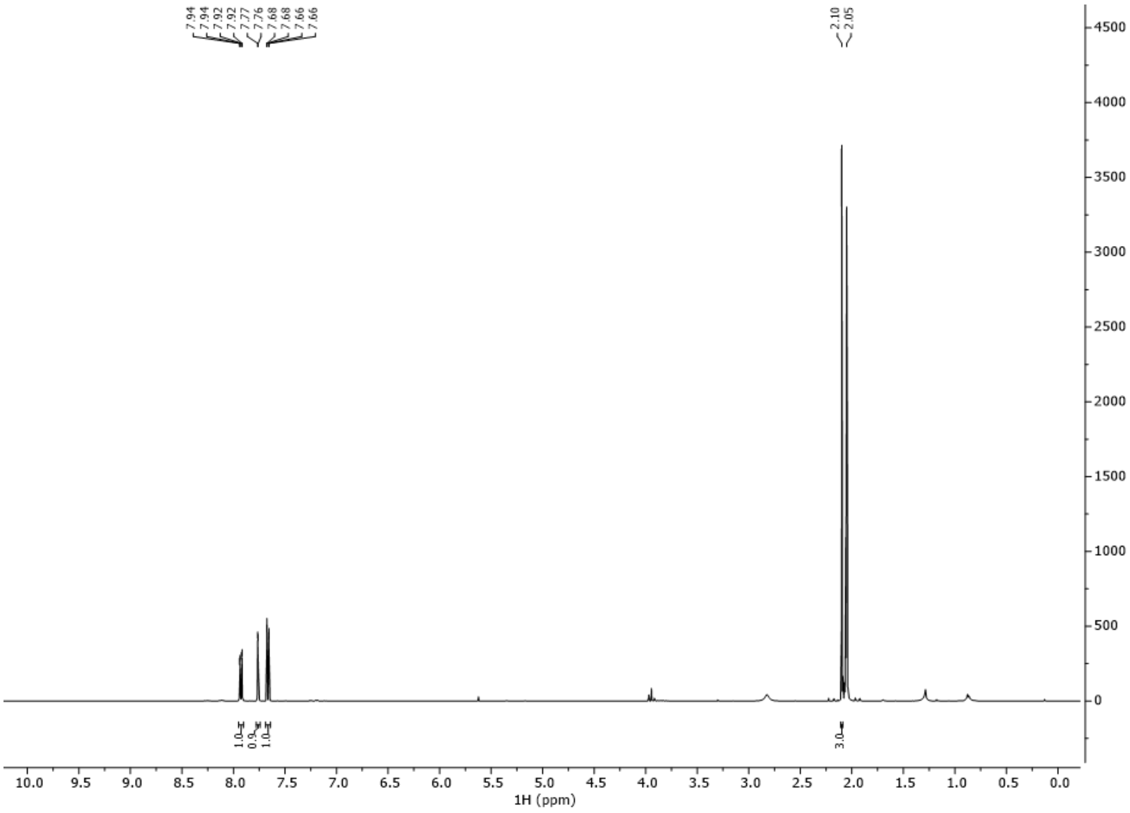

APT (acetone-d_6)

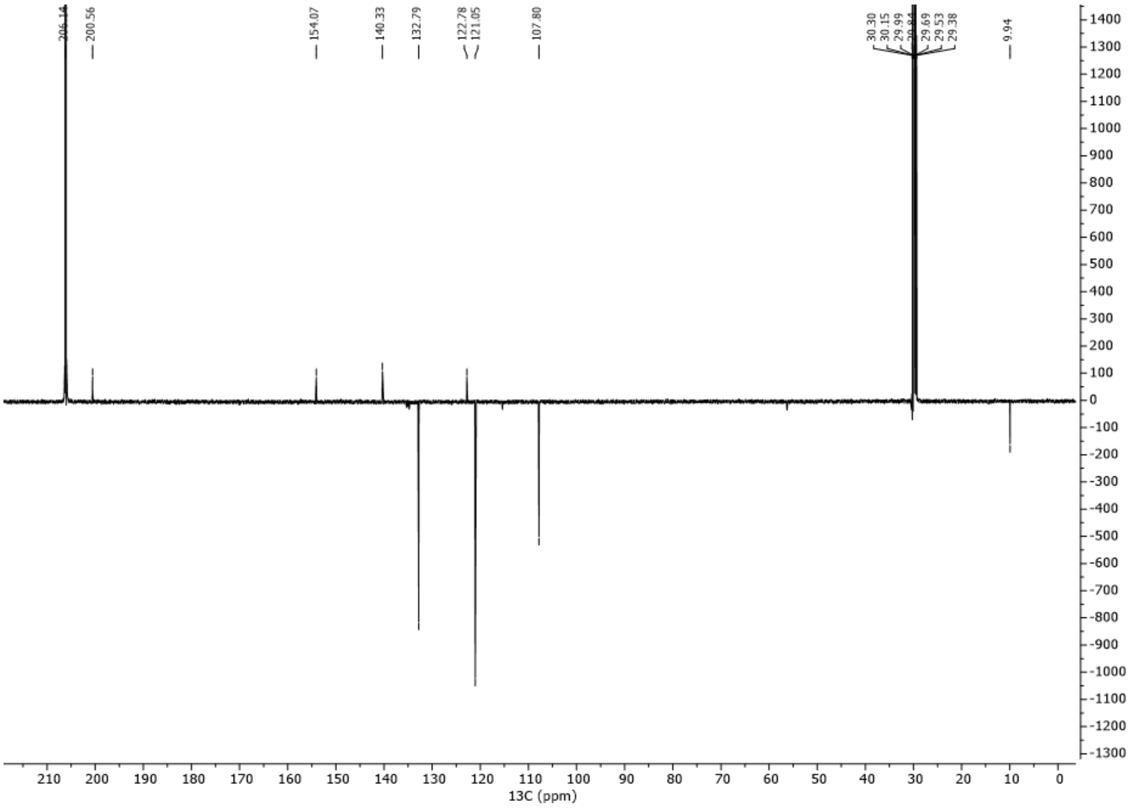

3,5-Dimethyl-2*H*-thiopyrano[3,4-*b*]furan-2-thione (**28**)

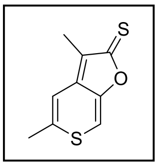

^{1}H NMR (acetone-d_6)

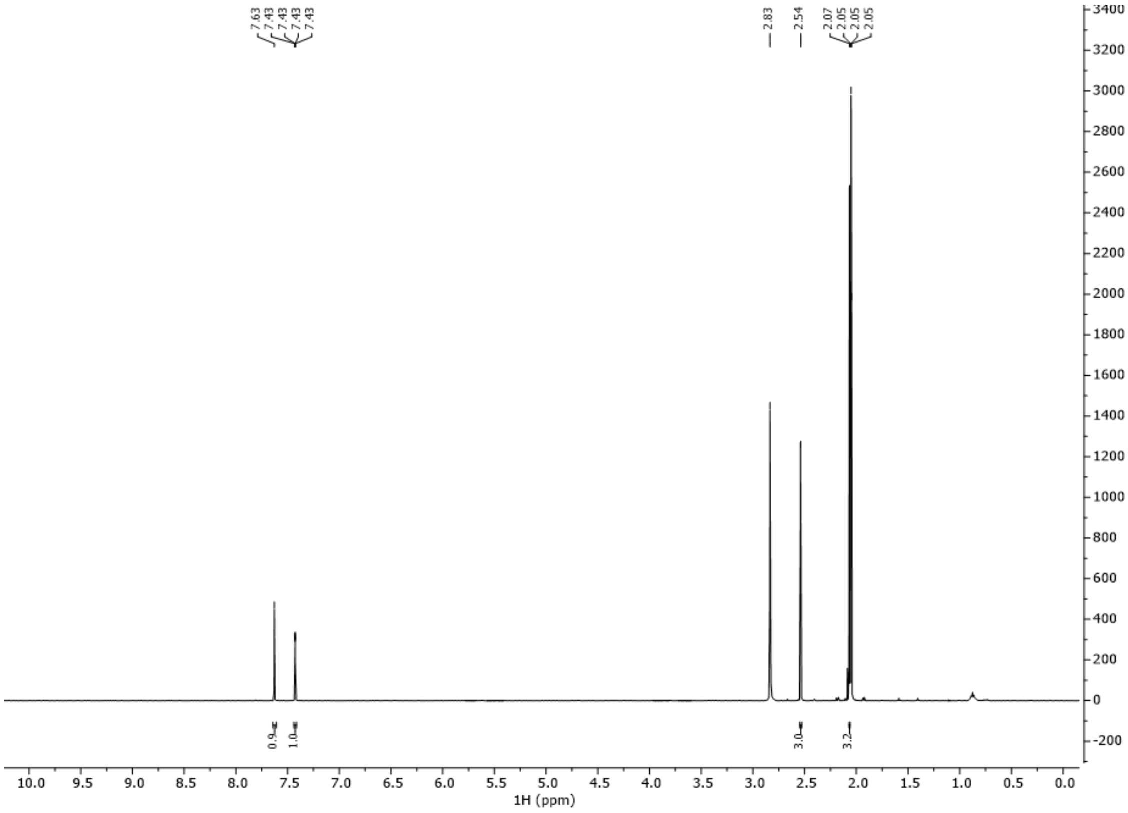

APT (acetone-d_6)

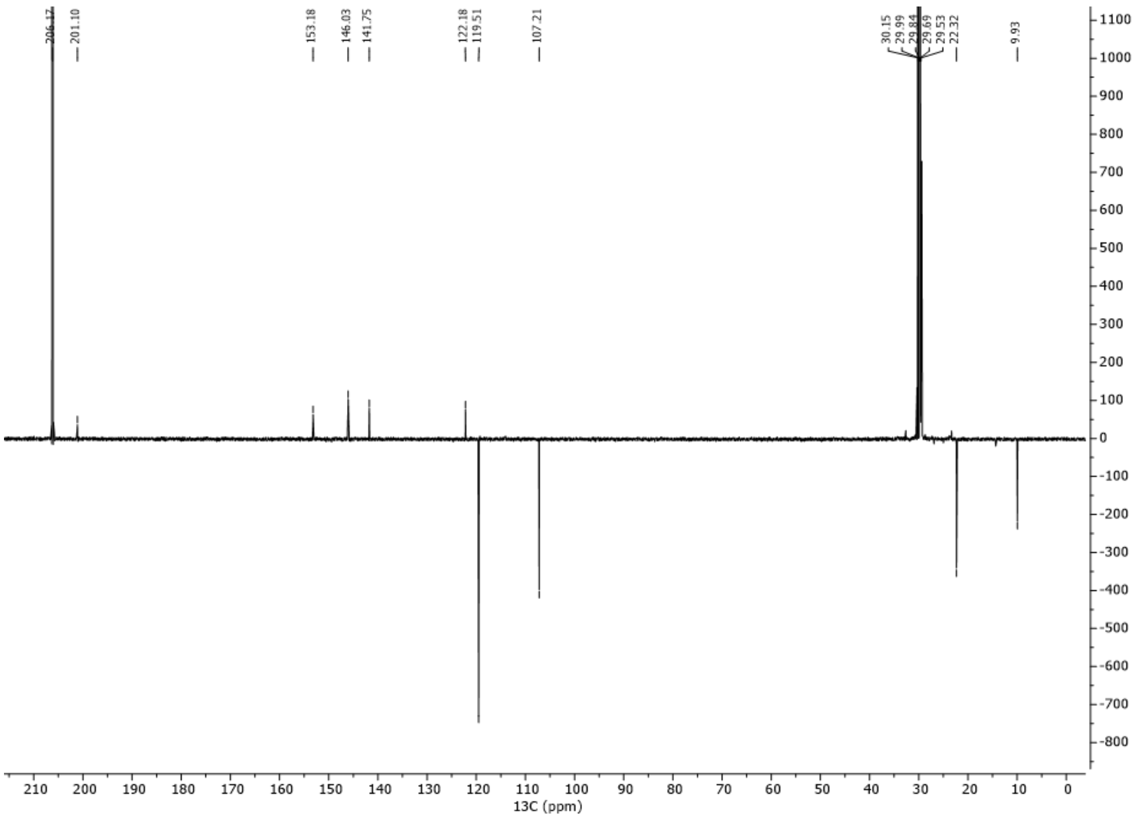

3,7-Dimethyl-2*H*-thiopyrano[3,4-*b*]furan-2-thione (**29**)

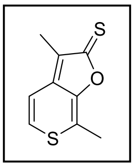

^{1}H NMR (acetone-d_6)

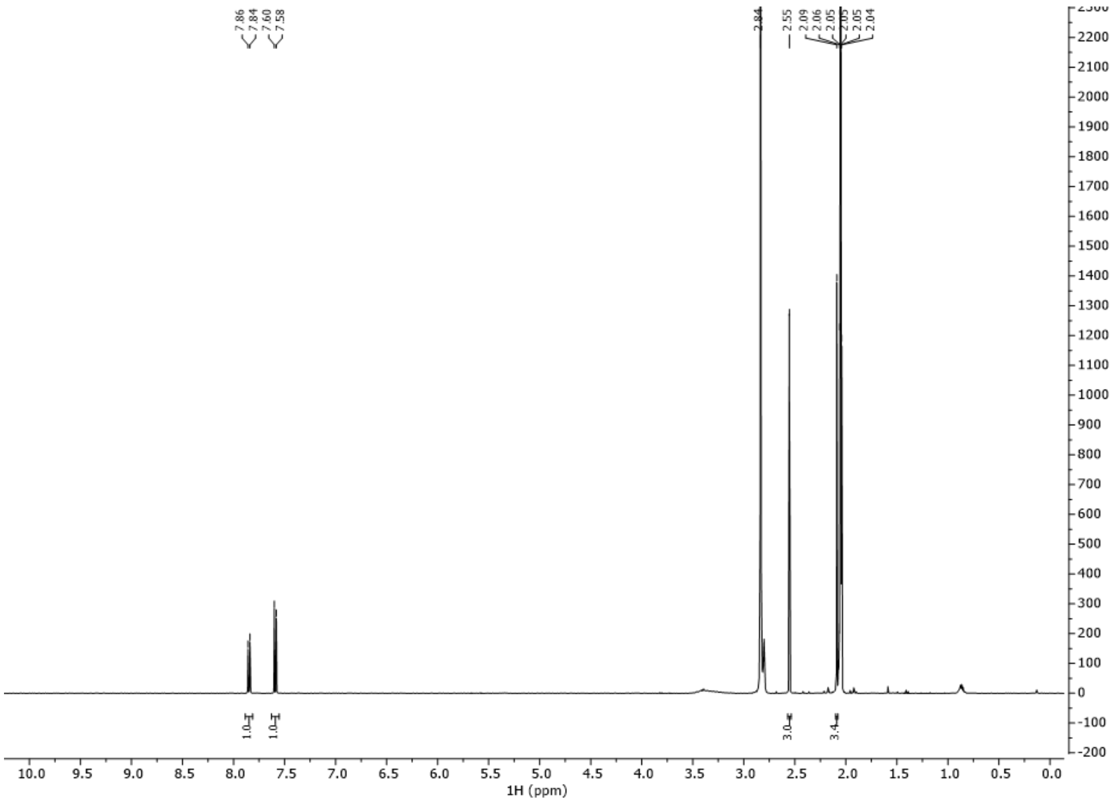

APT (acetone-d_6)

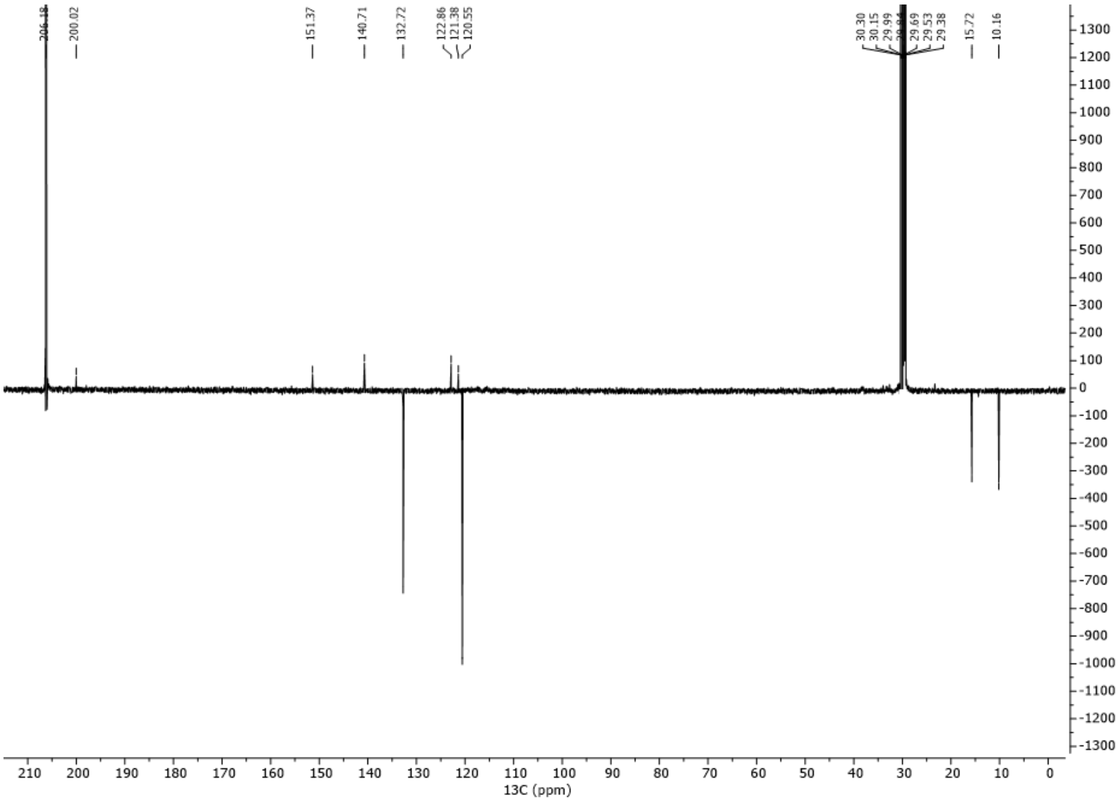

2*H*-Thiopyrano[3,4-*b*]furan-2-thione (**30**)

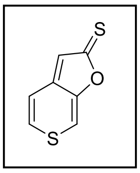

^{1}H NMR ($CDCl_3$)

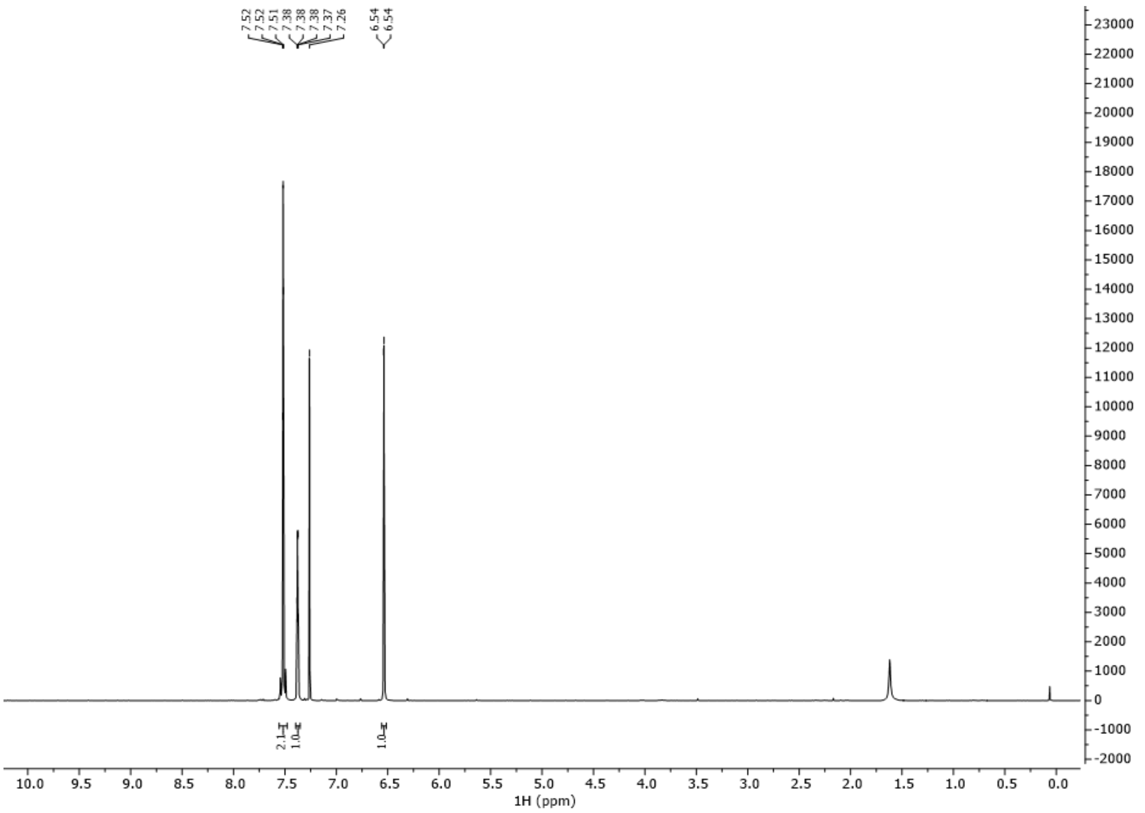

APT ($CDCl_3$)

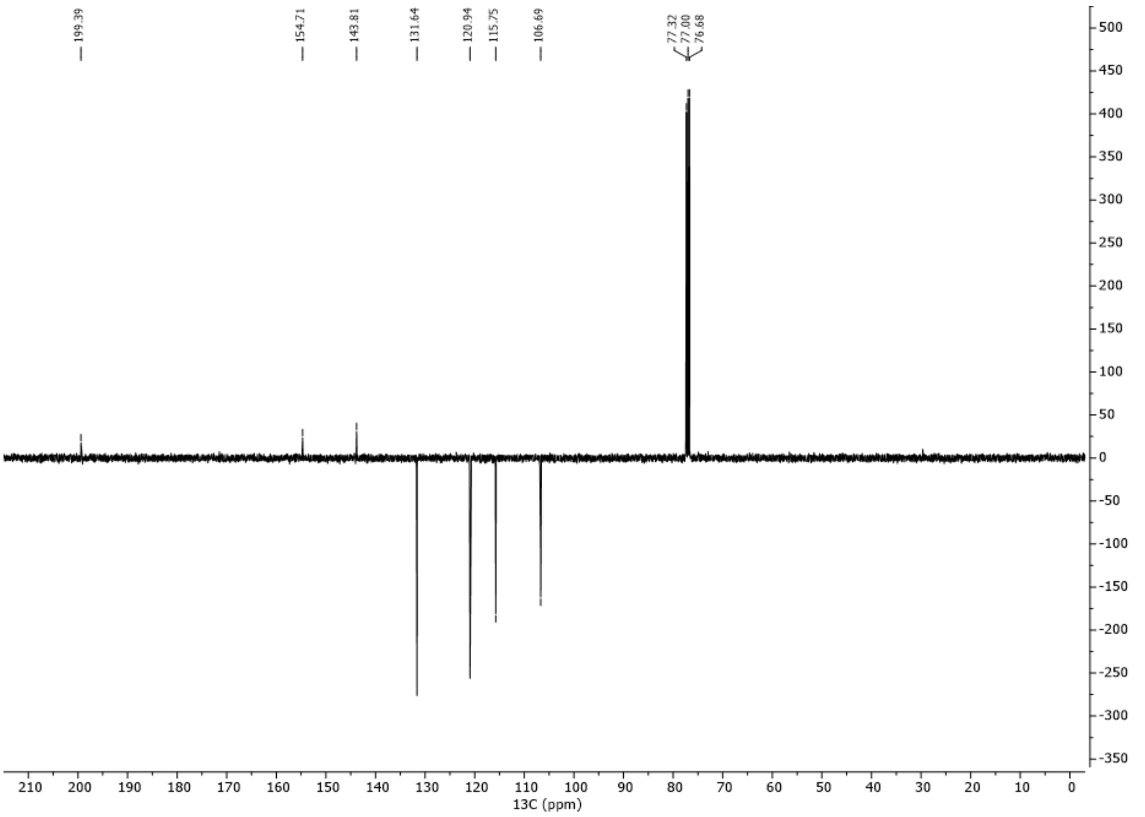

References

[1] Ellman, G. L.; Courtney, K. D.; Andres, V.; Featherstone, R. M. *Biochem. Pharmacol.* **1961**, *7*, 88-95. doi: 10.1016/0006-2952(61)90145-9

[2] Flematti, G. R.; Ghisalberti, E. L.; Dixon, K. W.; Trengove, R. D. *Tetrahedron Lett.* **2005**, *46*, 5719-5721. doi: 10.1016/j.tetlet.2005.06.077

[3] Flematti, G. R.; Goddard-Borger, E. D.; Merritt, D. J.; Ghisalberti, E. L.; Dixon, K. W.; Trengove, R. D. *J. Agric. Food Chem.* **2007**, *55*, 2189-2194. doi: 10.1021/jf0633241

[4] Goddard-Borger, E. D.; Ghisalberti, E. L.; Stick, R. V. *Eur. J. Org. Chem.* **2007**, 3925-3934. doi: 10.1002/ejoc.200700334

[5] Brayton, D.; Jacobsen, F. E.; Cohen, S. M.; Farmer, P. J. *Chem. Commun.* **2006**, 206-208. doi: 10.1039/B511966A

[6] Xavier, N. M.; Madeira, P. J. A.; Florêncio, M. H.; Rauter, A. P. *Eur. J. Org. Chem.* **2009**, 4983-4991. doi: 10.1002/ejoc.200900573
